# *Ngwevu intloko*: a new early sauropodomorph dinosaur from the Lower Jurassic Elliot Formation of South Africa and comments on cranial ontogeny in *Massospondylus carinatus*

**DOI:** 10.7717/peerj.7240

**Published:** 2019-08-05

**Authors:** Kimberley E.J. Chapelle, Paul M. Barrett, Jennifer Botha, Jonah N. Choiniere

**Affiliations:** 1 Evolutionary Studies Institute, University of the Witwatersrand, Johannesburg, South Africa; 2 School of Geosciences, University of the Witwatersrand, Johannesburg, South Africa; 3 Department of Earth Sciences, Natural History Museum, London, United Kingdom; 4 Department of Karoo Palaeontology, National Museum, Bloemfontein, South Africa; 5 Department of Zoology and Entomology, University of the Free State, Bloemfontein, South Africa

**Keywords:** Sauropodomorph, Elliot Formation, Ontogeny, *Ngwevu intloko*, *Massospondylus carinatus*, Stormberg, Osteohistology, Taxonomy, Computed tomography scans

## Abstract

Our knowledge of Early Jurassic palaeobiodiversity in the upper Elliot Formation of South Africa has increased markedly in recent years with the discovery of new fossils, re-assessments of previously collected material and a better understanding of Stormberg Group stratigraphy. Here, *Ngwevu intloko*, a new genus of upper Elliot basal sauropodomorph is named on the basis of a complete skull and partial skeleton (BP/1/4779) previously assigned to *Massospondylus carinatus*. It can be distinguished from all other basal sauropodomorphs by a combination of 16 cranial and six postcranial characters. The new species is compared to a small ontogenetic series of *M. carinatus* as well as to a range of closely related taxa. Taphonomic deformation, sexual dimorphism and ontogeny are rejected as possible explanations for the morphological differences present between BP/1/4779 and other taxa. Osteohistological examination reveals that BP/1/4779 had nearly reached adult size at the time of its death at a minimum age of 10 years.

## Introduction

*Massospondylus carinatus* is an iconic basal sauropodomorph dinosaur from the Lower Jurassic upper Elliot and Clarens formations of South Africa, Lesotho and from temporally equivalent formations in Zimbabwe (e.g. the Forest Sandstone Formation). It is the most common dinosaur in these deposits, and for more than 30 years was considered the only valid sauropodomorph taxon across its stratigraphic range in southern Africa (Owen, 1854; Ellenberger, Ellenberger & Ginsburg, 1970; Cooper, 1981; Kitching & Raath, 1984; Gow, 1990; Gow, Kitching & Raath, 1990; Knoll & Battail, 2001; Sues et al., 2004; Barrett & Yates, 2005; Knoll, 2005; Reisz et al., 2005, 2010; Yates & Barrett, 2010). *Massospondylus* was also established as the nominal taxon for the uppermost biozone in the Stormberg Supergroup, the *Massospondylus* Range Zone (Kitching & Raath, 1984).

However, the diversity of non-sauropodan sauropodomorph taxa in the upper Elliot and Clarens formations has increased in recent years with the description of several new taxa (and the re-dating of taxa previously considered to be from the lower Elliot Formation) including *Antetonitrus ingenipes*, *Massospondylus kaalae*, *Aardonyx celestae*, *Ignavusaurus rachelis*, *Arcusaurus pereirabdalorum*, *Pulanesaura eocollum* and *Ledumahadi mafube* (Yates & Kitching, 2003; Barrett, 2009; Knoll, 2010; Yates et al., 2010; Yates, Bonnan & Neveling, 2011; McPhee et al., 2015, 2018). Some of these were recognized through scrutiny of existing museum specimens, such as *M. kaalae* (Barrett, 2009) and *Antetonitrus ingenipes* (Yates & Kitching, 2003), while others resulted from new fieldwork, including *Pulanesaura eocollum* (McPhee et al., 2015). Ongoing work on the biostratigraphy of the Stormberg Group and its Assemblage Zones is also addressing taxon distributions and their relationships to lithostratigraphic boundaries and is being conducted in parallel with new chrono- and magnetostratigraphical dating in the lower and upper Elliot Formations (Bordy & Eriksson, 2015; Sciscio & Bordy, 2016; McPhee et al., 2017). For example, *Antetonitrus ingenipes* is now recognized as being Early Jurassic, rather than Late Triassic, in age (McPhee et al., 2017).

Hundreds of specimens have been attributed to *Massospondylus* since its description by Sir Richard Owen in 1854, including more than a dozen complete skulls (Owen, 1854; Kitching & Raath, 1984; Gow, 1990; Gow, Kitching & Raath, 1990; Smith & Kitching, 1997; Sues et al., 2004). Recently, *M. carinatus* has been the subject of focused anatomical research, which has provided amended cranial and postcranial diagnoses and comprehensive descriptions of type and referred material (Cooper, 1981; Barrett & Yates, 2005; Yates & Barrett, 2010; Chapelle & Choiniere, 2018; Barrett et al., 2019). Together, these studies have increased scrutiny on the comparative anatomy of early branching southern African sauropodomorphs more generally, inviting a reassessment of material referred to *Massospondylus*.

As part of our research group’s ongoing efforts to understand the palaeobiodiversity of the Elliot Formation, we reassessed some of the material previously referred to *M. carinatus*. Key differences were noted between the neotype and referred specimens of *M. carinatus* and another specimen, BP/1/4779, which has previously been referred to the taxon (Gow, Kitching & Raath, 1990; Sues et al., 2004). BP/1/4779, collected in 1978 by Prof. James W. Kitching, is a near-complete skeleton, including a strikingly well-preserved skull. It was referred to *M. carinatus* on the basis of general resemblance and provenance (Gow, Kitching & Raath, 1990) and on the presence of several cranial autapomorphies that had been proposed for the taxon (Sues et al., 2004), although the latter have now been shown to have limited taxonomic utility (Chapelle & Choiniere, 2018). Differences in cranial morphology between BP/1/4779 and other individuals were previously attributed to dorsoventral and anteroposterior compression during fossilization (Gow, Kitching & Raath, 1990; Sues et al., 2004). Here, we provide quantitative and qualitative morphological and osteohistological evidence that BP/1/4779 is not referable to *M. carinatus*, but represents a near-adult specimen of a new massospondylid taxon.

## Methods

BP/1/4779 is compared to the holotype of *M. carinatus* and two other specimens that can confidently be referred to that taxon on the basis of shared cranial and postcranial characters (BP/1/4934, BP/1/5241, BP/1/4376) (Gow, Kitching & Raath, 1990; Sues et al., 2004; Chapelle & Choiniere, 2018; Barrett et al., 2019). The majority of skull comparisons were carried out using BP/1/5241 as a standard for *M. carinatus*, as this specimen has been the most thoroughly described and is only 0.17 times larger than BP/1/4779 (based on femoral circumferences) (Gow, Kitching & Raath, 1990; Sues et al., 2004; Chapelle & Choiniere, 2018). BP/1/4376 is used for comparisons with a juvenile *M. carinatus*; BP/1/4934 is the neotype of *M. carinatus* and another example of a larger adult skull. These three specimens represent a partial ontogenetic series and are, therefore, also useful for testing whether developmental stage might be responsible for the morphological differences observed between BP/1/4779 and other massospondylids. We also compared BP/1/4779 to the only other currently valid species of *Massospondylus*, *M. kaalae*, which is represented by a single, incomplete and partially articulated skull (SAM-PK-K1325: Barrett, 2009). Comparisons were also drawn between BP/1/4779 and other massospondylids and putative members of the clade, notably *Adeopapposaurus mognai*, *Coloradisaurus brevis*, *Ignavusaurus rachelis*, *Leyesaurus marayensis*, *Lufengosaurus huenei* and *Sarahsaurus aurifontanalis* as well as another Elliot Formation taxon, *Arcusaurus pereirabdalorum*. Comparisons were made on the basis of personal observations and published accounts (see File S1 for specimen information) (Barrett, Upchurch & Wang, 2005; Barrett, 2009; Martínez, 2009; Knoll, 2010; Apaldetti et al., 2011, 2014; Yates, Bonnan & Neveling, 2011; Marsh & Rowe, 2018).

BP/1/4779 was scanned at the Wits Microfocus X-ray computed tomography (CT) facility of the Palaeosciences Centre at the University of the Witwatersrand. The facility uses a Nikon Metrology XTH 225/320 LC dual source industrial CT system. The X-ray characteristics were set at 120 kV and 250 mA, and a 1.2 mm thick copper filter applied. The resulting data dimensions were as follows: 1,998 × 1,998 × 315 with a Voxel Size of 0.09459 mm. Skulls and individual cranial bones were segmented in VG Studio MAX 3.2 (Volume Graphics, Heidelberg, Germany) and used for cranial linear measurements. Postcranial linear measurements were taken directly from the specimen using digital callipers and a tailors’ tape.

BP/1/4779 was scored into a recently revised character matrix (Chapelle & Choiniere, 2018) (see File S2 for matrix). The matrix, comprising 142 cranial characters and 240 postcranial characters was used to score BP/1/4779 in Mesquite v3.31 (Maddison & Maddison, 2015). Phylogenetic analyses were then performed in TNT v1.5 (Goloboff & Catalano, 2016). The first step of the analysis was the ‘Stabilize Consensus’ option in the ‘New Technology Search’ using sectorial searches and tree fusing, with the consensus stabilized five times. Resulting trees were then submitted to a ‘traditional search’, swapping using tree bisection-reconnection (TBR). A strict consensus of these trees was determined. These trees were also subjected to a final round of analysis, saving sub-optimal trees up to 10 steps longer and stopping when maxtrees hit 10,000. Absolute Bremer supports were then calculated from these 10,000 trees.

A destructive sampling permit was acquired (permit number 2643) from the South African Heritage Resources Agency in order to section several bones of BP/1/4779 for osteohistological analysis. All osteohistological sections were produced at the National Museum, Bloemfontein following standard methods (Botha-Brink, Soares & Martinelli, 2018). Two-to-three-centimetre-long sections of the midshaft of the right humerus and of the left femur were cut using a Dremel^®^ tool. These were then embedded under vacuum in Struers EpoFix^®^ resin and left to dry for 36 hours. The embedded bones were cut into 1.5 mm-thick cross-sections using a Struers Accutom-100^®^. Thick sections were adhered to five mm-thick glass slides with EpoFix^®^ resin, then ground to a thickness of only a few microns using the Struers Accutom-100^®^. Rendering was carried out under normal, polarized and cross-polarized (CPL) light, using polarizing microscopes (Nikon Eclipse Ci-POL) equipped with a digital camera (DS-Fi3), in NIS-Elements 4.5 (Nikon Corp., Tokyo, Japan). Stitched images of complete slide scans were assembled using NIS-Elements.

The electronic version of this article in portable document format will represent a published work according to the International Commission on Zoological Nomenclature (ICZN), and hence the new names contained in the electronic version are effectively published under that Code from the electronic edition alone. This published work and the nomenclatural acts it contains have been registered in ZooBank, the online registration system for the ICZN. The ZooBank Life Science Identifiers (LSIDs) can be resolved and the associated information viewed through any standard web browser by appending the LSID to the prefix http://zoobank.org/. The LSID for this publication is: [urn:lsid:zoobank.org:pub:839E7CB8-82D6-47FF-B348-43ED9106D995]. The online version of this work is archived and available from the following digital repositories: PeerJ, PubMed Central and CLOCKSS.

## Results

SYSTEMATIC PALAEONTOLOGY
DINOSAURIA Owen, 1842
SAURISCHIA Seeley, 1887
SAUROPODOMORPHA von Huene, 1932
*Ngwevu intloko* gen. et sp. nov.

**Holotype:** BP/1/4779, a partially complete skeleton including skull.

**Locality and horizon:** Tevrede (1077) Farm, Fouriesberg District, Free State Province, South Africa. Uppermost upper Elliot Formation, Stormberg Group (?Hettangian–?Sinemurian: Lower Jurassic). Only one other specimen in the ESI collections was recovered from this locality, comprising of medium-sized postcranial remains of an unidentified sauropodomorph.

**Etymology:** From Xhosa, ‘ngwevu’ meaning grey and ‘intloko’ meaning head. Pronounced ‘Ng-g’where-voo in-tloh-koh’. In reference to the affectionate nickname, ‘grey skull’, that had been given to BP/1/4779 by many of the scientists who worked on it previously.

**Diagnosis:** *Ngwevu intloko* can be distinguished from other basal sauropodomorphs on the basis of a unique character combination comprising 16 cranial and six postcranial characters, including one autapomorphy (indicated by an asterisk): a cranium that is wider than it is high (cranium width to height ratio of ± 1.7); a wide skull relative to its length (skull width to length ratio ± 0.6); a robust postorbital (anteroposterior length of jugal ramus base to skull length ratio 0.07); frontals that are wider than they are long (fused frontals width to length ratio 1.15); proportionally wide parietals (fused parietals width to length ratio 0.7); squamosal rami of the parietal diverge from each other at an angle >105°; paroccipital processes of the exoccipitals diverge from each other at an angle >105°; a semilunate supraoccipital that is wider than it is high (character 82, state 1); the absence of a ridge between the basisphenoid and basioccipital basal tubera components (character 91, state 0); the ventral margin of the basal tubera being ventral to the proximal base of the basipterygoid processes (character 98, state 1); proportionally long pterygoids (pterygoid length to skull length ratio 0.53); an anterodorsally oriented palate; a longitudinal ridge on the posterolateral surface of the jugal*; a broad ‘U’-shaped jaw (character 115, state 1); a maximum dorsoventral height to anteroposterior length of the dentary ratio >0.2 (character 118, state 1); procumbent dentary teeth (character 132, state 1); no more than 14 vertebrae between the cervicodorsal transition and sacral vertebrae (character 168, state 1); no dorsosacral vertebrae (character 199, state 0); a poorly defined fossa on the distal flexor surface of humerus (character 232, state 1); a distal transverse width to proximodistal length of the humerus ratio <0.33 (character 233, state 0); a metacarpal I proximal width to proximodistal width ratio between 0.65 and 0.8 (character 249, state 1); and a weakly bent femur (character 302, state 1).

## Morphological Descriptions and Comparisons

### General observations and skull openings

As noted by previous authors, BP/1/4779 shares many similarities with *M. carinatus*, both cranially and postcranially, so the following description highlights differences between these specimens rather than repeating anatomical details that have been figured and described in detail elsewhere (Gow, Kitching & Raath, 1990; Sues et al., 2004; Chapelle & Choiniere, 2018; Barrett et al., 2019). A full detailed anatomical description will be published in future by the authors.

**Figure 1:**
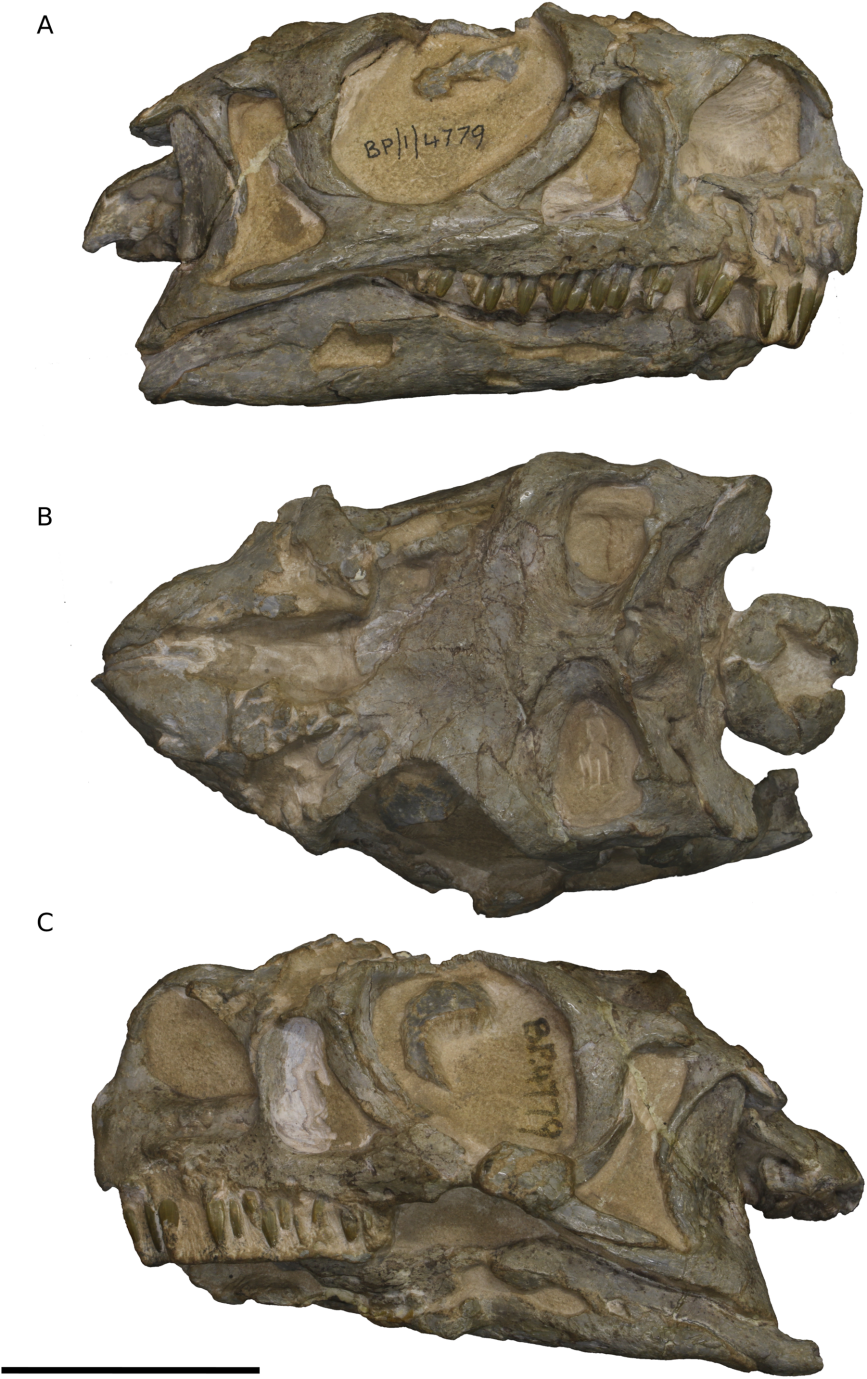
Photographs of the skull of BP/1/4779. (A) Right lateral view. (B) Dorsal view. (C) Left lateral view. Scale bar represents 10 mm. Photographs by Kimberley E.J. Chapelle.

**Figure 2:**
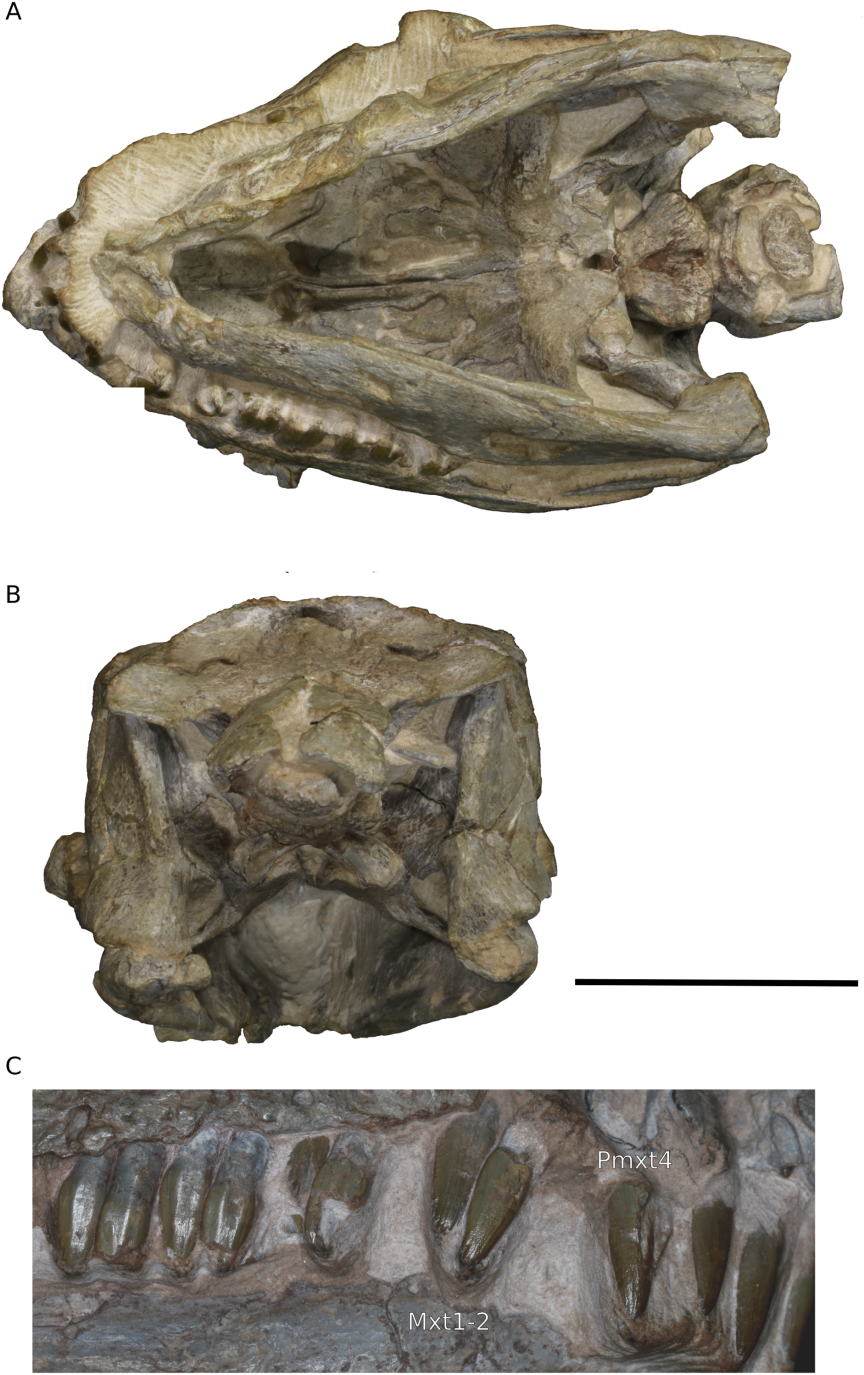
Photographs of the skull of BP/1/4779. (A) Ventral view. (B) Posterior view. (C) Closeup of anterior maxillary teeth and premaxillary teeth. Scale bar represents 10 mm. Abbreviations: Mxt, maxillary teeth; Pmxt, premaxillary teeth. Photographs by Kimberley E.J. Chapelle.

**Figure 3:**
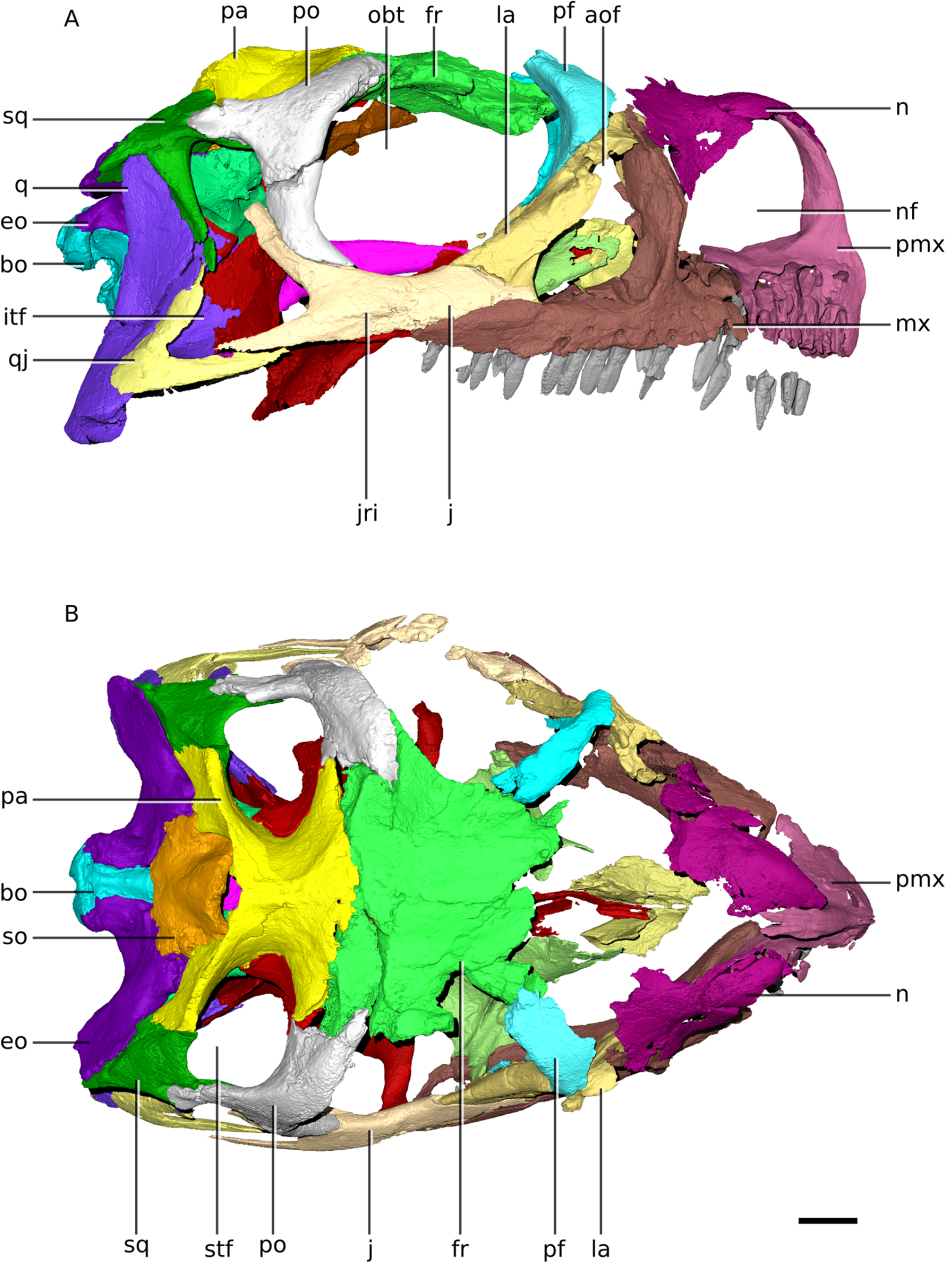
Digital reconstruction of the cranium of BP/1/4779. (A) Right lateral view. (B) Dorsal view. Scale bar represents 10 mm. Abbreviations: aof, antorbital fenestra; bo, basioccipital; eo, exoccipital; fr, frontal; itf, infratemporal fenestra; j, jugal; jri, jugal ridge; la, lacrimal; mx, maxilla; n, nasal; nf, narial fenestra; obt, orbit; pa, parietal; pf, prefrontal; pmx, premaxilla; po, postorbital; q, quadrate; qj, quadratojugal; so, supraoccipital; sq, squamosal; stf, supratemporal fenestra.

**Figure 4:**
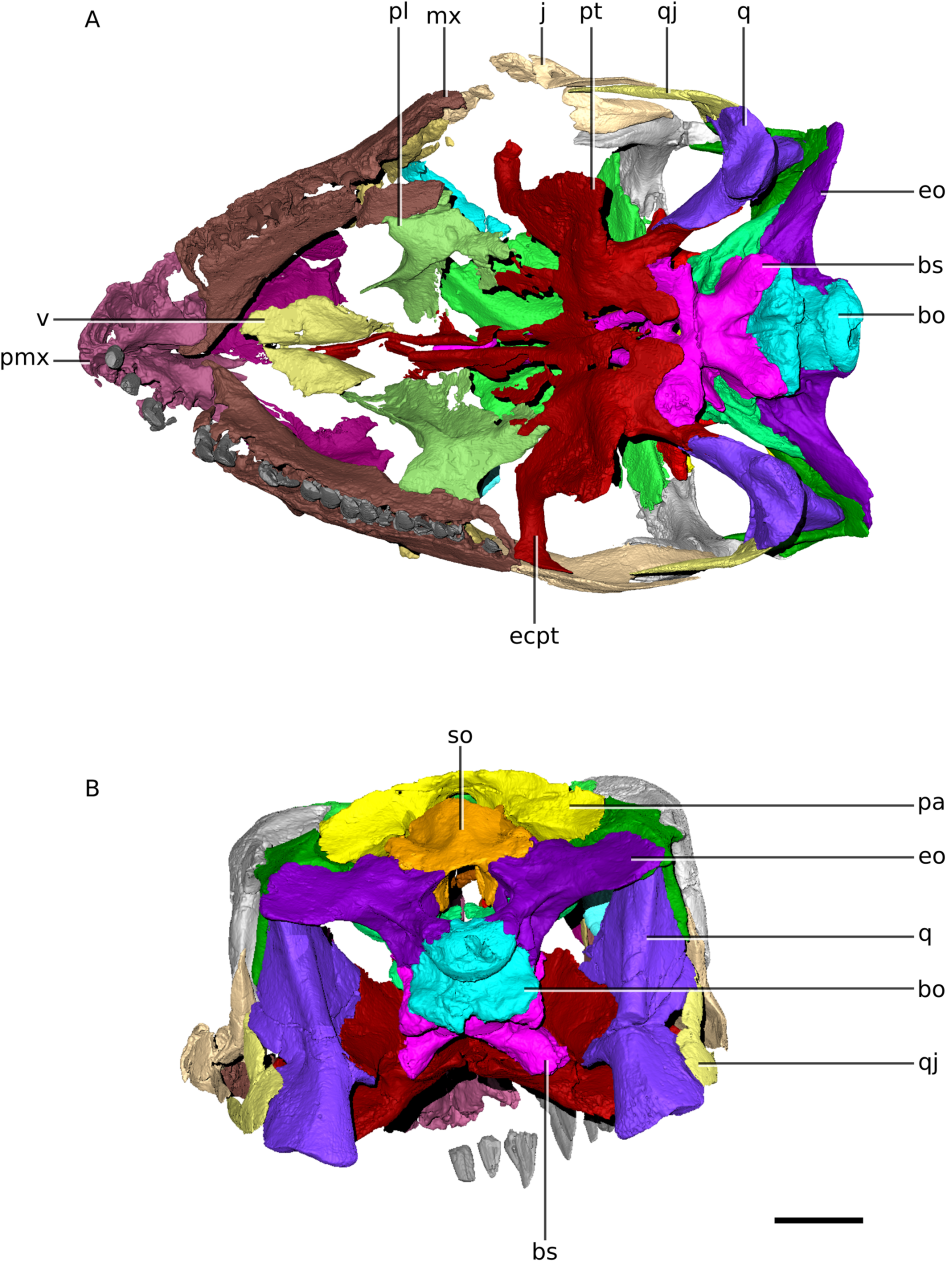
Digital reconstruction of the skull of BP/1/4779. (A) Ventral view. (B) Posterior view. Scale bar represents 10 mm. Abbreviations: bo, basioccipital; bs, basisphenoid; ecpt, ectopterygoid; eo, exoccipital; j, jugal; mx, maxilla; pa, parietal; pl, palatine; pmx, premaxilla; pt, pterygoid; qj, quadratojugal; q, quadrate; so, supraoccipital; v, vomer.

**Figure 5:**
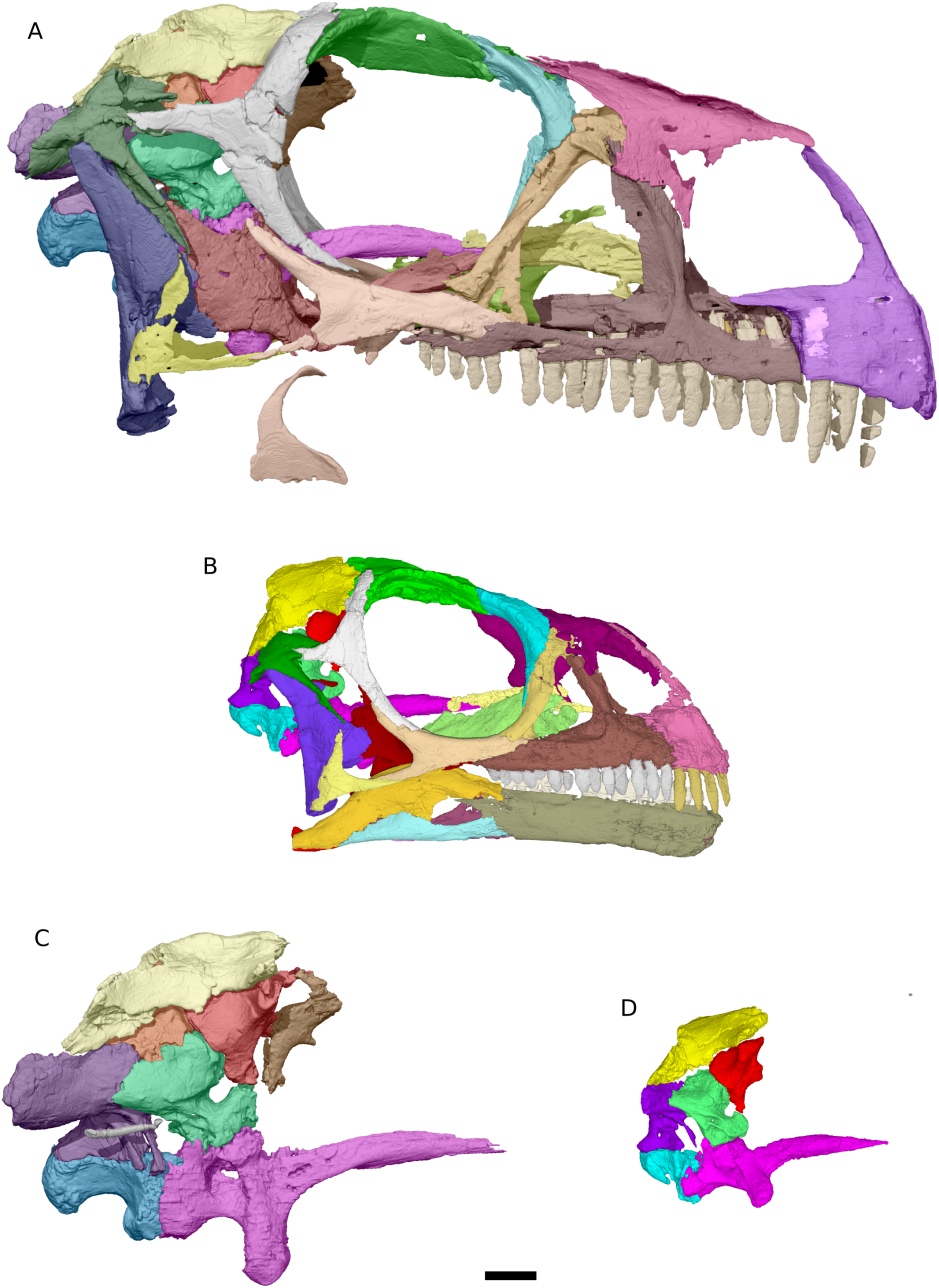
Digital reconstruction of the skull and braincase of *M. carinatus* adult and juvenile. (A) Adult skull BP/1/5241 in right lateral view. (B) Juvenile skull BP/1/4376 in right lateral view. (C) Adult braincase BP/1/5241 in right lateral view. (D) Juvenile braincase BP/1/4376 in right lateral view. Scale bar represents 10 mm.

Although there is some slight anterodorsal distortion of the skull, as shown by the shape of the orbit and the angle of the quadrate (which slopes posteroventrally at 12.5° from the vertical), there does not appear to be any dorsoventral crushing and BP/1/4779 is otherwise well preserved and symmetrical (Figs. 1–5).

In general, *Ngwevu* differs from *M. carinatus* in having much more robust cranial bones, as shown by the various bone measurement to skull length ratios (Table 1). This is especially marked with respect to the jugal maxillary ramus dorsoventral height, the postorbital jugal ramus anteroposterior length, and the squamosal quadratojugal ramus anteroposterior length.

**Table 1:** Comparative measurements of BP/4779 and a representative ontogenetic series of *M. carinatus*. All measurements are in millimeters.

Measurements	BP/1/4779	BP/1/4934	BP/1/5241	BP/1/4376
Femoral circumference	119	214	145	--
Skull maximum anteroposterior length	133.87	218.05	187.04	96.12
Skull greatest mediolateral width	81.29	76.86	59.79	39.16
Skull dorsoventral height at orbit excluding mandible	47.61	71.89	65.62	36.91
Skull width/height	1.71	1.07	0.91	1.06
Skull width/length	0.61	0.35	0.32	0.41
Skull height/length	0.36	0.33	0.35	0.38
Supratemporal fenestra anteroposterior length	18.46	26.24	27.31	14.81
Supratemporal fenestra mediolateral width	25.82	10.69	13.46	9.23
Supratemporal fenestra length/width	0.71	2.45	2.03	1.60
Anteroposterior length of prefrontal frontal ramus	22.38	48.49	26.65	18.25
Dorsoventral height of the prefrontal frontal ramus	7.44	14.95	8.89	6.00
External naris maximum anteroposterior length	31.88	33.79	33.3	15.04
Naris length/skull length	0.24	0.18	0.15	0.16
Orbit maximum anteroposterior length	41.13	61.09	49.59	33.56
Frontal body maximum mediolateral width	42.76	--	30.77	--
Frontal maximum anteroposterior length	37.1	--	44.09	--
Frontal length/width	0.87	--	1.43	--
Parietal maximum anteroposterior length	27.7	43.01	46.82	24.13
Parietal body maximum mediolateral width	19.43	19.86	19.7	21.52
Parietal angle between squamosal rami	134.1	87	67	--
Parietal length/width	1.43	2.17	2.38	1.12
Antorbital fossa maximum anteroposterior length	20.78	41.62	36.4	16.27
Postorbital jugal process base anteroposterior length	13.49	12.64	10.43	4.6
Postorbital squamosal ramus base anteroposterior length	8.72	9.96	8.53	4.84
Postorbital jugal ramus base/skull length	0.10	0.06	0.06	0.05
Jugal dorsoventral height under orbit	9.94	12.05	9.58	4.99
Squamosal quadratojugal ramus base anteroposterior length	7.6	10.12	6.2	4.23
Squamosal quadratojugal ramus dorsoventral height	22.46	32.44	30.17	17.85
Squamosal quadratojugal ramus dorsoventral height/anteroposterior base length	2.96	3.21	4.87	4.22
Squamosal angle between parietal ramus and postorbital ramus	85°	50°	50°	85°
Jugal height/skull length	0.07	0.06	0.05	0.05
Squamosal quadratojugal ramus base length/skull length	0.06	0.05	0.03	0.04
Angle between quadratojugal rami	40.7	62.8	61	67.3
Angle between quadratojugal and pterygoid rami of the quadrate	95°	--	22°	29°
Quadrate maximum mediolateral width of the condylar region/quadrate maximum dorsoventral height	0.30	--	0.24	0.21
Vomer anteroposterior length	24.9	--	58.1	--
Pterygoid anteroposterior length	72	--	49.04	--
Pterygoid mediolateral width of main body/anteroposterior length of the main body	0.53	--	0.3	--
pterygoid length/skull length	0.54	--	0.26	--
Vomer length/pterygoid length	0.35	--	1.18	--
Vomer length/skull length	0.19	--	0.31	--
Orbitosphenoid main body anteroposterior length/dorsoventral height	0.09	--	0.31	--
Basisphenoid cultriform process base dorsoventral height	10.85	9.03	12.89	5.09
Basisphenoid cultriform process base mediolateral width	10.57	9.42	11.56	3.67
Basisphenoid cultriform process anteroposterior length	40.23	44.77	59.13	27.34
Basisphenoid cultriform process base height/length	0.27	0.20	0.22	0.19
Basisphenoid cultriform process base width/length	0.26	0.21	0.20	0.13
Supraoccipital mediolateral width in posterior view	24.98	33	24.94	--
Supraoccipital dorsoventral height in posterior view	17.30	35.13	25.08	--
Supraoccipital width/length in posterior view	1.44	0.94	0.99	--

**Note:**

Double dashes indicate unobtainable measurements.

Overall skull proportions differ markedly between *Ngwevu* and those of *M. carinatus* (Table 1). The maximum mediolateral skull width to dorsoventral skull height ratio is 1.7 in *Ngwevu* and approximately 1.0 in *M. carinatus* (in both adults and juveniles). The maximum mediolateral skull width to maximum anteroposterior skull length ratio is 0.6 in *Ngwevu* but between 0.32 and 0.41 in *M. carinatus* (in adults and juveniles, respectively). The maximum dorsoventral skull height to maximum anteroposterior skull length ratio does not differ much, however, and is 0.36 in *Ngwevu* and between 0.33 and 0.38 in *M. carinatus* (in adults and juveniles, respectively).

The proportions of the skull openings in *Ngwevu* also differ from those in *M. carinatus*, which is congruent with the overall differences in skull shape. The supratemporal fenestra of *Ngwevu* is mediolaterally wider than it is anteroposteriorly long, unlike that of *M. carinatus* (length to width ratio of 0.71 in *Ngwevu*; between 2.5 and 1.6 in *M. carinatus* adults and juvenile, respectively). *Adeopapposaurus* also has a supratemporal fenestra that is longer than wide. The external naris of *Ngwevu* is also proportionally longer than in *M. carinatus* (external naris length to skull length ratio 0.24 in *Ngwevu* and between 0.16 and 0.18 in *M. carinatus*).

### Premaxilla

**Figure 6:**
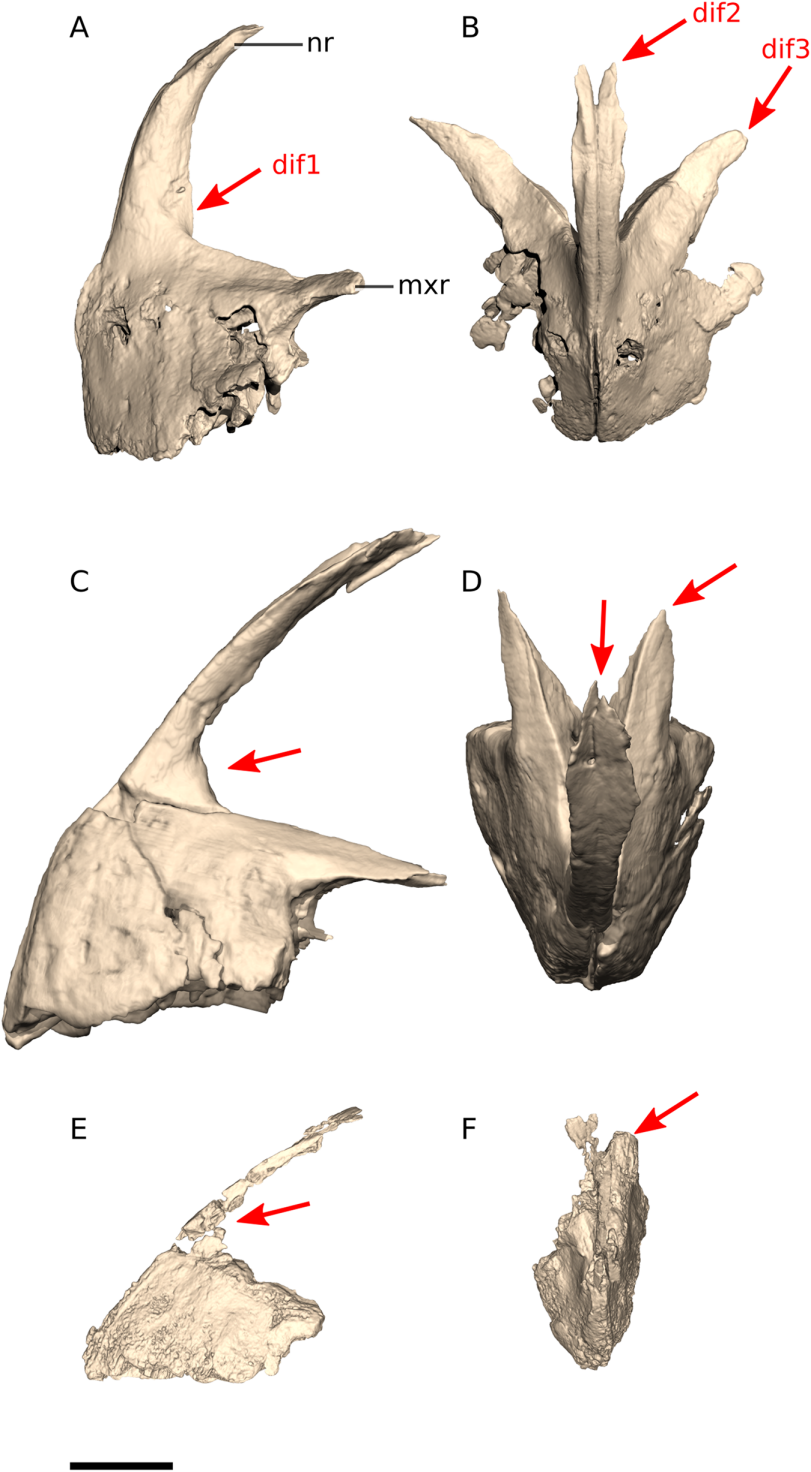
Digital reconstruction of the premaxillae of BP/1/4779 and *M. carinatus* adult and juvenile. (A) BP/1/4779 in left lateral view. (B) BP/1/4779 in dorsal view. (C) Adult BP/1/5241 in left lateral view. (D) Adult BP/1/5241 in dorsal view. (E) Juvenile BP/1/4376 in left lateral view. (F) Juvenile BP/1/4376 in dorsal view. Scale bar represents 10 mm. Red arrows point at areas of main differences: dif1, the nasal ramus extends almost perpendicularly from the main body in *Ngwevu* and extends posteriorly only at its distal end whereas in *M. carinatus* the nasal ramus extends posterodorsally from the main body in a smooth continuous arc; dif2, the nasal ramus of *Ngwevu* is forked distally whereas that of *M. carinatus* adults tapers to a point; dif3, in *Ngwevu* the maxillary rami of the premaxillae extend more laterally than in *M. carinatus*. Abbreviations: mxr, maxillary ramus; nr, nasal ramus.

In *Ngwevu*, the nasal ramus of the premaxilla extends almost perpendicularly from the main body and extends posteriorly only at its distal end whereas in *M. carinatus* (adults and juvenile), the nasal ramus extends posterodorsally from the main body in a smooth continuous arc, forming a rounded anteroventral corner to the external naris (Fig. 6). The nasal ramus of *Ngwevu* is forked distally whereas that of *M. carinatus* adults tapers to a point (this is difficult to confirm in the juvenile due to poor preservation).

In *Ngwevu* the maxillary rami of the premaxillae extend more strongly laterally than in *M. carinatus*, forming an angle of 88° between them. These rami have concave lateral margins and convex medial margins in dorsal view, so that the snout is broad and ‘U’-shaped. By contrast, in *M. carinatus* adults the maxillary rami diverge at an angle of approximately 30° (not determinable in the juvenile) and have straight margins in dorsal view, giving a ‘V’-shaped outline. *Arcusaurus* is similar in morphology to *Ngwevu* and the medial shelf on the dorsal margin of the lateral surface of the premaxilla described in *Arcusaurus* appears to correspond to the lateral surface of the maxillary ramus, although it is poorly preserved. This shelf forms the dorsal margin of the articular surface for the anterior process of the maxilla. This is seen in *M. carinatus* and *Ngwevu*.

### Maxilla

**Figure 7:**
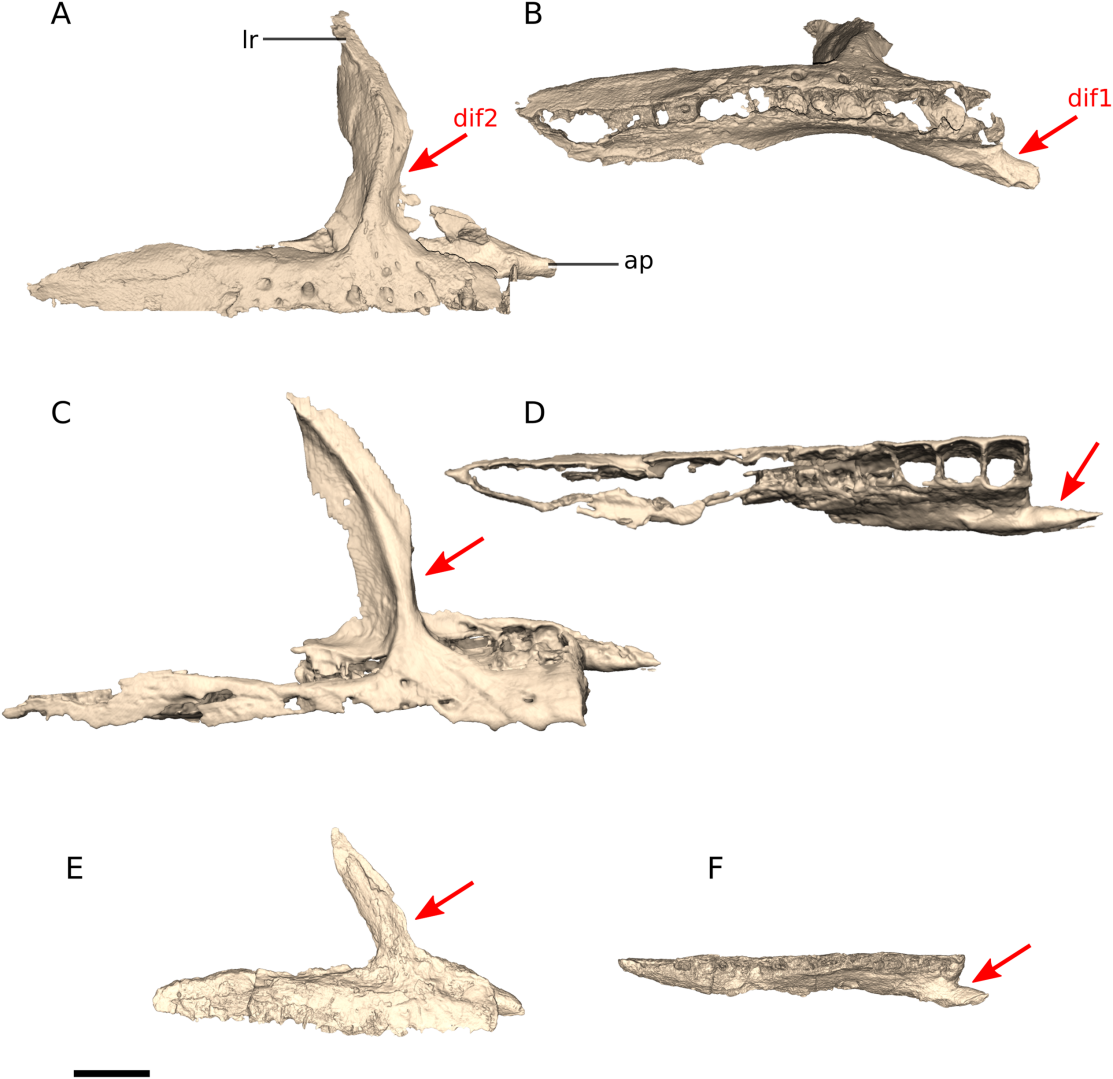
Digital reconstruction of the maxillae of BP/1/4779 and *M. carinatus* adult and juvenile. (A) BP/1/4779 in right lateral view. (B) BP/1/4779 in ventral view. (C) Adult BP/1/5241 in right lateral view. (D) Adult BP/1/5241 in ventral view. (E) Juvenile BP/1/4376 in left lateral view. (F) Juvenile BP/1/4376 in ventral view (BP/1/4376 mirrored to facilitate comparisons). Scale bar represents 10 mm. Red arrows point at areas of main differences: dif1, the anterior process extends anteromedially in *Ngwevu* whereas in *M. carinatus* it extends strictly anteriorly; dif2, in *Ngwevu* the ventral half of the lacrimal ramus extends anterodorsally, but changes in orientation so that its dorsal half extends posterodorsally, whereas in *M. carinatus* (juveniles and adults) this ramus extends posterodorsally along its entire length. Abbreviations: ap, anterior process; lr, lacrimal ramus.

In *Ngwevu*, the anterior process that articulates with the premaxilla extends anteromedially, giving the entire maxilla a concave medial margin and a convex lateral margin in dorsal/ventral view. In *M. carinatus* (adults and juveniles), however, the maxilla has linear medial and slightly convex lateral margins and the anterior process extends strictly anteriorly (Fig. 7).

In *Ngwevu* the ventral half of the lacrimal ramus of the maxilla extends anterodorsally, but changes in orientation so that its dorsal half extends posterodorsally, whereas in *M. carinatus* (juveniles and adults) this ramus extends posterodorsally along its entire length. Although this difference might be the result of the slight distortion mentioned previously, this seems unlikely as post-mortem crushing might also lead to cracking or changes in bone fibre orientation, but neither of these are observed.

### Nasal

In *Ngwevu*, the nasal has a more prominent concave dorsal margin in lateral view than that in *M. carinatus* (Figs. 1A and 3A), *Coloradisaurus* and *Adeopapposaurus*. This feature is similar to that in *Lufengosaurus huenei. Ngwevu* does not possess the anteroventral process of the nasal that is present in *Arcusaurus*. There appears to be an anteriorly-facing small notch on the posterior margin of the maxillary ramus of the right nasal in *Ngwevu*, although this could be due to preservation and cannot be confirmed on the left side due to breakage.

### Prefrontal

In *Ngwevu*, the lacrimal ramus of the prefrontal extends ventrally to a point that is equal to approximately 0.5 that of the dorsoventral height of the lacrimal, which is similar to the condition in *M. carinatus* (adult and juvenile) (Fig. 3A).

### Lacrimal

**Figure 8:**
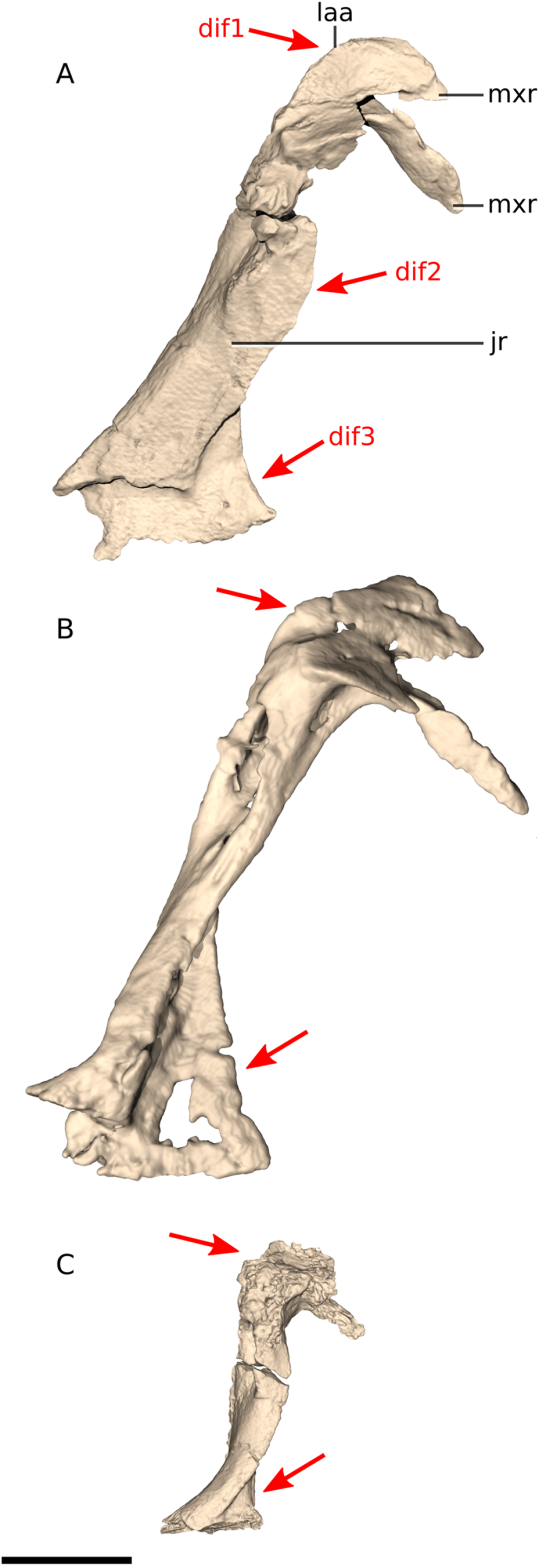
Digital reconstruction of the lacrimals of BP/1/4779 and *M. carinatus* adult and juvenile. (A) BP/1/4779 in right lateral view. (B) Adult BP/1/5241 in right lateral view. (C) Juvenile BP/1/4376 right lateral view. Scale bar represents 10 mm. Red arrows point at areas of main differences: dif1, *Ngwevu* has a very rounded, convex lacrimal angle in lateral/medial view in comparison to *M. carinatus* juvenile and adults; dif2, *Ngwevu* has an anteriorly flaring lateral surface of the lacrimal shaft, not present in *M. carinatus*; dif3, the dorsoventral height of the distal expansion is proportionally lower in *Ngwevu* and *M. carinatus* juvenile than in *M. carinatus* adult. Abbreviations: jr, jugal ramus; laa, lacrimal angle; mxr, maxillary ramus.

*Ngwevu* has a very rounded, convex lacrimal angle in lateral/medial view in comparison to *M. carinatus* juvenile and adults (Fig. 8). *Ngwevu* also has an anteriorly flaring lateral surface of the lacrimal shaft, giving the latter a convex anterior margin. This is not present in *M. carinatus* (adult or juvenile). *Adeopapposaurus* appears to have a similar feature on the anterior margin of its lacrimal, but it is much more pronounced and resembles a distinct boss rather than an anterior flaring of the anterior margin. In *Ngwevu*, the convex anterior margin of the lacrimals gives the antorbital fenestra a crescentic shape with the anterior and posterior margins of the opening being subparallel. This crescentic shape is also present, although less pronounced, in *Lufengosaurus huenei*.

In *Ngwevu* the jugal ramus of the lacrimal expands anteroposteriorly as it extends distally. The anterior margin of this expanded portion is excavated to form the posterior corner of the antorbital fossa. The dorsoventral height of this distal expansion is equivalent to 0.27 that of the dorsoventral height of the entire lacrimal. In *M. carinatus* BP/1/5241, by contrast, this expansion occupies 0.44 the complete dorsoventral height of the lacrimal. The juvenile *M. carinatus* specimen is more similar to *Ngwevu* with regards to the distal lacrimal morphology and *Lufengosaurus huenei, Adeopapposaurus* and *M. kaalae* also have a similar distal lacrimal. In *Sarahsaurus*, there is no expansion of the distal lacrimal.

### Postorbital

The postorbital of *Ngwevu* is very robust in comparison to that of *M. carinatus* (Fig. 5). The mediolateral width of the jugal ramus is less than its anteroposterior length at midshaft as in *Lufengosaurus huenei*, *M. kaalae*, *Sarahsaurus* and *Coloradisaurus* but unlike *M. carinatus*, *Adeopapposaurus, Ignavusaurus* and *Leyesaurus* where the mediolateral width of the jugal ramus is more than its anteroposterior length at midshaft.

The distal end of the jugal ramus of the postorbital extends farther anteriorly than the rest of the ramus, giving it a kinked anterior margin rather than the smooth circular margin seen in *M. carinatus*. This feature is also seen in *Sarahsaurus* but is likely due to taphonomic distortion in both this taxon and *Ngwevu*.

*Ngwevu* and adult *M. carinatus* specimens have a pronounced orbital rim that projects more laterally that the squamosal ramus of the postorbital in dorsal view. The juvenile *M. carinatus* has a less distinct postorbital rim that is level with the lateral surface of the squamosal ramus (posterior process) in dorsal view. *Lufengosaurus huenei* also has a robust postorbital and pronounced orbital rim that is similar to *Ngwevu*, whereas *Adeopapposaurus*, *Arcusaurus*, *Ignavusaurus*, *Leyesaurus* and *M. kaalae* have a more gracile morphology that is more similar to that of *M. carinatus. Coloradisaurus* and *Sarahsaurus* appear to have robust postorbitals also, although this is difficult to confirm due to deformation and preservation.

### Squamosal

In dorsal view, the parietal ramus and the postorbital ramus in *Ngwevu* are separated by a wide ‘U’-shaped notch (forming an angle of approximately 85°) (Figs. 1B and 3B). The anterior margin of this notch forms the posterolateral corner of the supratemporal fenestra. This is similar to the condition in the *M. carinatus* juvenile, *Sarahsaurus*, *Coloradisaurus* and *Lufengosaurus huenei*. In *M. carinatus* adults, these rami are separated by a more acute angle of approximately 50°.

In *M. carinatus* (BP/1/5241 and BP/1/4376), the quadrate ramus is relatively elongate and >4.0 times its anteroposterior length at the base as in *Adeopapposaurus* and *Coloradisaurus*. By contrast, in *Ngwevu*, BP/1/4934, *Lufengosaurus huenei* and *Sarahsaurus* this ratio is <4.0 (Figs. 1A and 3A).

### Jugal

*Ngwevu* possesses an anteroposteriorly-oriented ridge on the posterior half of the lateral surface of the jugal main body that is absent in all other massospondylids, and is regarded herein as a possible autapomorphy of the taxon. This ridge is more conspicuous on the right side (Figs. 1A and 3A).

In *Ngwevu*, the postorbital ramus of the jugal is proportionally short compared to that of *M. carinatus*. In *M. carinatus*, the ratio of the proximodistal length of this ramus to the anteroposterior length of the main body of the jugal decreases during ontogeny (postorbital ramus proximodistal length to jugal main body anteroposterior length ratio of 0.50 in *Ngwevu*, 0.52 in BP/1/4934, 0.60 in BP/1/5241 and 0.69 in BP/1/4376). This process does not taper to a point on the right side of *Ngwevu* but it does on the left side suggesting that the right postorbital ramus is missing its distal end.

In *Ngwevu* as well as in *M. carinatus*, the postorbital ramus of the jugal forms a 60–70° angle with the quadratojugal ramus. In *Adeopapposaurus*, the postorbital ramus of the jugal extends posterodorsally and is linear, forming a 40° angle with the quadratojugal ramus. In *Sarahsaurus* and *Lufengosaurus huenei* the postorbital ramus also extends posterodorsally, is linear, and forms a 60° angle and a 47° angle, respectively.

### Quadratojugal

Both quadratojugals are well preserved in *Ngwevu* (Figs. 1 and 3). The left one has small cracks at the base of the jugal and squamosal rami, however, this does not affect the shape and it is not disarticulated. The angle formed by the squamosal and jugal rami of the quadratojugal is approximately 41° in *Ngwevu*, between 61° and 63° in adult *M. carinatus* specimens and 67° in the *M. carinatus* juvenile. This angle therefore decreases slightly during ontogeny in *M. carinatus*. However, *Ngwevu* has a much more acute angle than the adults, although this difference might have been slightly exaggerated by distortion. In *Adeopapposaurus* an angle of approximately 66° separates the two quadratojugal rami, similar to *M. carinatus*, whereas this angle is 45° in *Lufengosaurus huenei*, and thus more similar to that of *Ngwevu*.

### Quadrate

**Figure 9:**
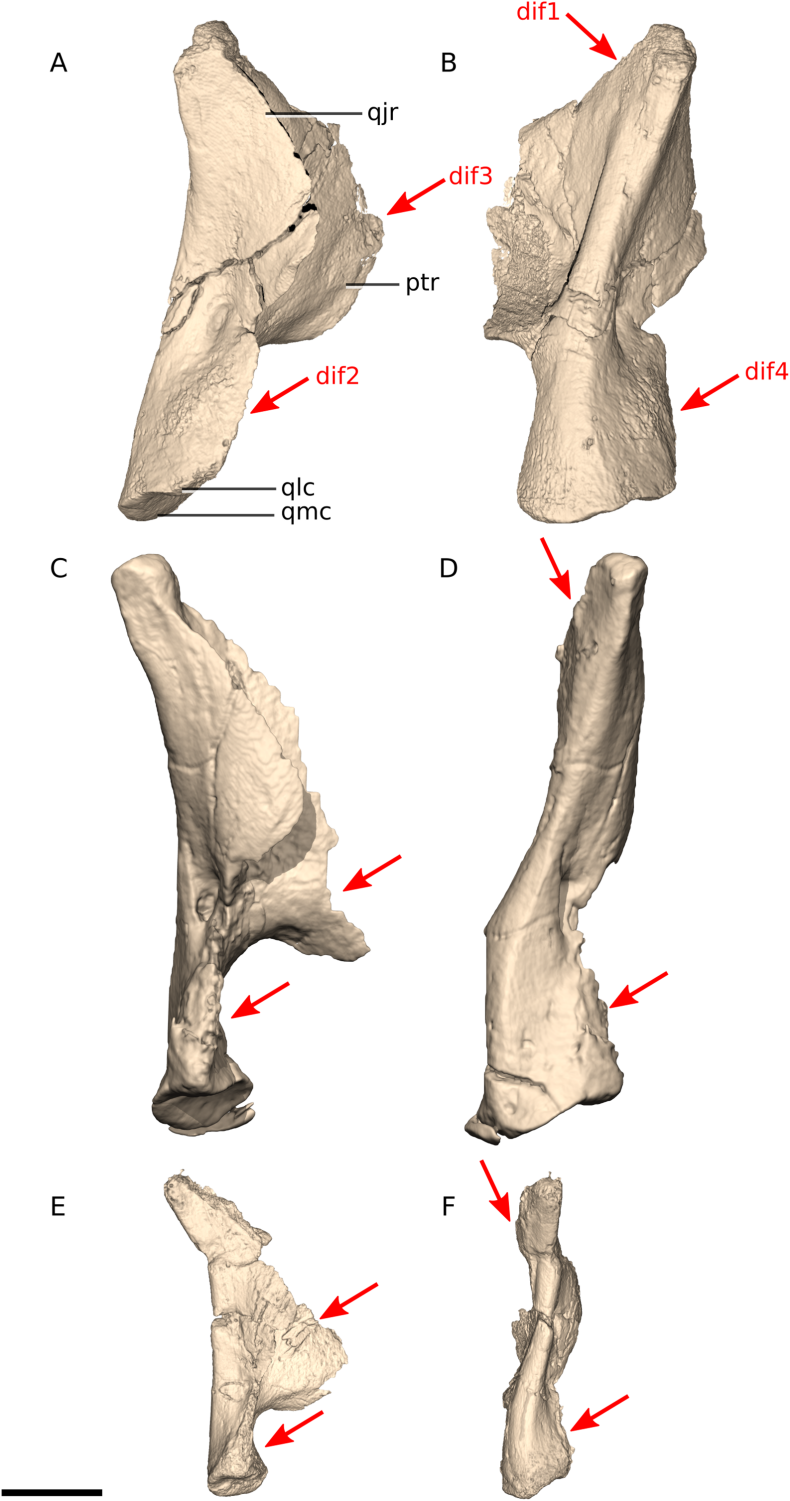
Digital reconstruction of the quadrates of BP/1/4779 and *M. carinatus* adult and juvenile. (A) BP/1/4779 in left lateral view. (B) BP/1/4779 in posterior view (BP/1/4779 mirrored to facilitate comparisons). (C) Adult BP/1/5241 in right lateral view. (D) Adult BP/1/5241 in posterior view. (E) Juvenile BP/1/4376 in right lateral view. (F) Juvenile BP/1/4376 in posterior view. Scale bar represents 10 mm. Red arrows point at areas of main differences: dif1, in *Ngwevu*, the pterygoid and quadratojugal rami form a wider angle than in *M. carinatus*; dif2, in *Ngwevu*, the anterior margin of the ventral portion of the quadrate is convex whereas it is concave in *M. carinatus*; dif3, the quadratojugal and pterygoid rami have a near semi-circular outline in lateral view in *Ngwevu*, whereas they are triangular in shape in *M. carinatus*; dif4 the condylar region is proportionally broader mediolaterally in *Ngwevu* than in *M.* carinatus. Abbreviations: ptr, pterygoid ramus; qjr, quadratojugal ramus; qlc, quadrate lateral condyle; qmc, quadrate medial condyle.

In *Ngwevu*, the pterygoid and quadratojugal rami form a 95° angle whereas in *M. carinatus* specimens this angle is 22° and 29° in adults (BP/1/5241) and juveniles, respectively (Fig. 9). BP/1/4934 appears to have a broader angle separating the rami (approximately 90°), but only a small portion of the quadratojugal ramus is preserved so this is difficult to confirm. The angle between the pterygoid and quadratojugal rami also appears to be more acute in *Adeopapposaurus* but this is difficult to confirm from the published images. In *Coloradisaurus* and *Lufengosaurus huenei* this angle is nearly 90°.

In *Ngwevu*, the anterior margin of the ventral portion of the quadrate, beneath the quadratojugal ramus, is convex in lateral view whereas it is concave in *M. carinatus* (in both adults and juveniles). The quadratojugal and pterygoid rami have a near semi-circular outline in lateral view in *Ngwevu*, whereas they are triangular in shape in *M. carinatus*. The pterygoid ramus dorsoventral height to quadrate dorsoventral ratio is 0.62 in *Ngwevu* and approximately 0.60 and 0.63 in adult and juvenile *M. carinatus* specimens, respectively. This is also seen in *Coloradisaurus*. In *Adeopapposaurus* and *Coloradisaurus*, however, the pterygoid ramus is proportionally taller dorsoventrally and has a ratio of more than 0.7 with respect to the height of the quadrate.

*Adeopapposaurus* and *Leyesaurus* have a strongly concave posterior quadrate margin that differs from that in *Ngwevu*, which is almost linear. In posterior view, *Leyesaurus* has a sinusoidal quadrate morphology with the medial margin convex in its ventral half and concave dorsally. This morphology differs from that in *Ngwevu*, *M. carinatus* and *Adeopapposaurus* in which the dorsal two-thirds of the quadrate are dorsolaterally oriented and the ventral third is dorsoventrally oriented. The morphology of the quadrate in posterior view is difficult to confirm in *Coloradisaurus* (although it looks slightly sinusoidal), *Sarahsaurus* and *Lufengosaurus huenei* due to preservation.

Finally, the condylar region is proportionally broader mediolaterally in *Ngwevu* than in *M. carinatus* (maximum mediolateral width of the condylar region to quadrate maximum dorsoventral height ratio of 0.3 in *Ngwevu* and 0.24 in the *M. carinatus* adult (BP/1/5241) and 0.21 in *M carinatus* juvenile). In the *M. carinatus* adult BP/1/4934, the condyle mediolateral width to quadrate dorsoventral height ratio appears to be similar to that of *Ngwevu*, but this may be exaggerated by the numerous cracks present in the quadrates.

### Frontal

The frontals are fused in *Ngwevu* and are wider than they are long (mediolateral width to anteroposterior length ratio of 1.15) (Fig. 3B). In *M. carinatus* adults (BP/1/5241), the opposite is true (mediolateral width to anteroposterior length ratio of 0.70). Unfortunately, the frontals of BP/1/4376 are too badly preserved to confirm the state in juvenile *M. carinatus*. Similarly, the frontals of BP/1/4934 are also poorly preserved. *Adeopapposaurus* has a similar condition to *M. carinatus* adults with a mediolateral width to anteroposterior length ratio of 0.59. *Sarahsaurus* has fused frontals that are almost as wide as they are long with a ratio of 0.98. In *Lufengosaurus huenei* this ratio is 1.15, similar to *Ngwevu*.

In *Ngwevu*, the supratemporal fossa excavates the posterior portion of the frontals, forming a scarp-like rim. This is also observed in *Sarahsaurus*, *Lufengosaurus huenei* and *Adeopapposaurus*, but is absent or poorly developed in *M. carinatus*, *Coloradisaurus* and *Leyesaurus.*

### Vomer

**Figure 10:**
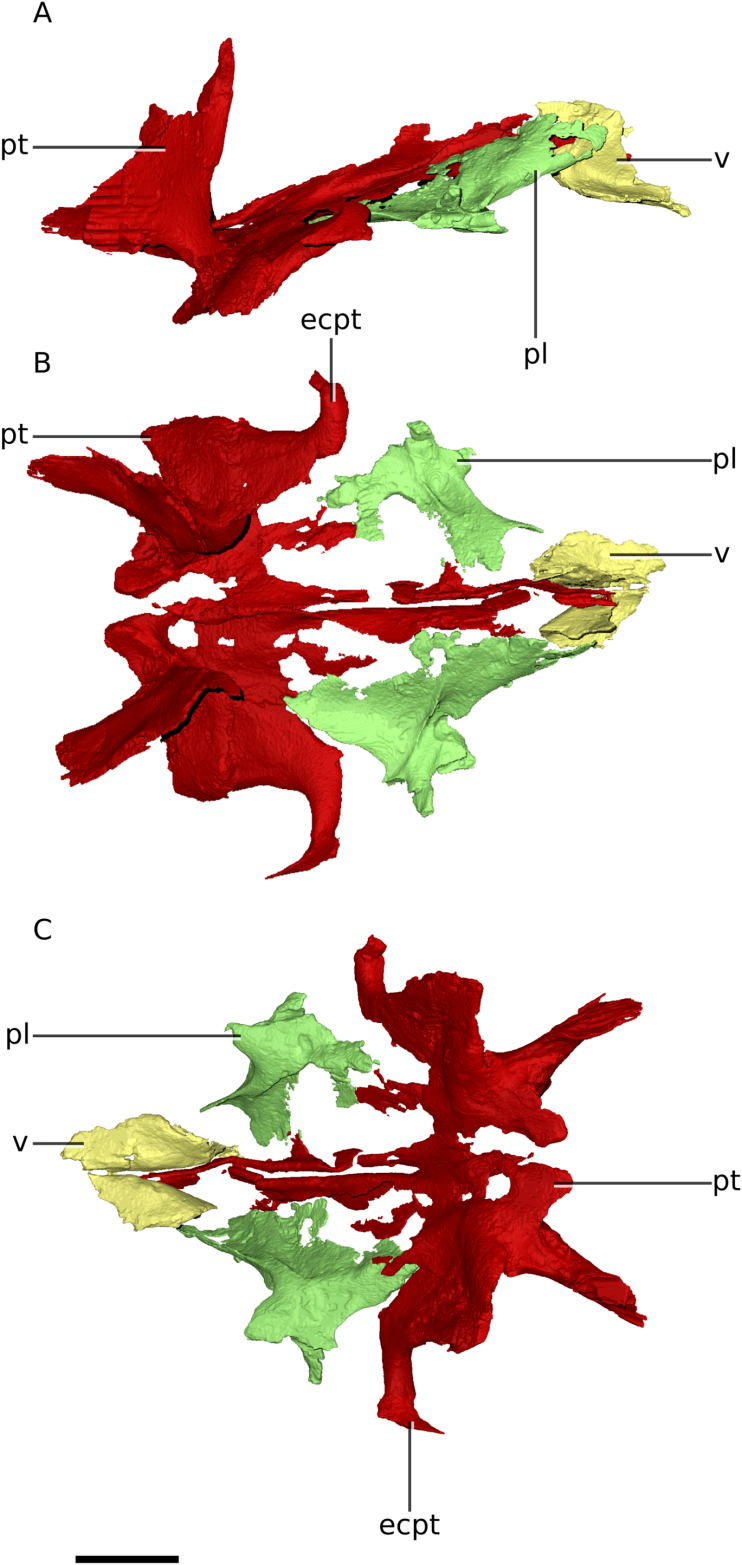
Digital reconstruction of the palate of BP/1/4779. (A) Right lateral view. (B) Dorsal view. (C) Ventral view. Scale bar represents 10 mm. Abbreviations: ecpt, ectopterygoid; pl, palatine; pt, pterygoid; v, vomer.

In *Ngwevu*, as preserved, the anteroposterior length of the fused vomers is 24.9 mm, but if this measurement is extended to the premaxillary contact, this gives a maximum length of approximately 29.2 mm (Figs. 2A, 4A and 10). The latter provides a vomer anteroposterior length to skull length ratio of 0.22 and a vomer anteroposterior length to pterygoid palatine ramus length ratio of 0.55. In *M. carinatus* (BP/1/5241), the vomer to skull length ratio is of 0.31 and the vomers are longer than the pterygoid palatine ramus, with a ratio of 1.5. The arched morphology of the vomers described in *M. carinatus* cannot be confirmed in *Ngwevu* as the anterior portion is not preserved. However, the preserved proximal portions of the vomers in *Ngwevu* slope gently anteroventrally whereas in *M. carinatus* they rise anterodorsally. In *Adeopapposaurus*, the anteroposterior vomer length to pterygoid palatine ramus length ratio is 0.65.

### Pterygoid

In comparison with adults of *M. carinatus*, the fused pterygoids of *Ngwevu* are mediolaterally broad relative to their anteroposterior length (mediolateral width to anteroposterior length ratio of the main body of 0.53 in *Ngwevu* and 0.30 in BP/1/5241) (Figs. 2A, 4A and 10). *Ngwevu* possesses hook-like medial processes that wrap around the basipterygoid processes of the basisphenoid as also occur in *M. carinatus*, but in *Ngwevu* these form a 70° angle with the ventral margin of the posterior portion of the pterygoid whereas in *M. carinatus* this angle is approximately 40°.

### Ectopterygoid

The ectopterygoids of *Ngwevu* are fused with the pterygoids and difficult to differentiate (Figs. 2A, 4A and 10). In *M. carinatus* (BP/1/5241) they are disarticulated from the rest of the skull. The jugal rami of the ectopterygoids are strongly recurved and taper to a point as they extend distally, similar to *M. carinatus*. In *Adeopapposaurus*, the ectopterygoid jugal ramus is not as recurved and does not taper distally, but is blunt ended.

### Palatine

The general morphology of the palatine does not differ greatly from that of *M. carinatus*, but its proportions and orientation do (Figs. 2A, 4A and 10). *Ngwevu* has a proportionally broad palatine with a mediolateral width to anteroposterior length ratio of approximately 0.47, whereas *M. carinatus* has a more gracile palatine with a ratio of 0.25.

In lateral view, the long axis of the palatine of *Ngwevu* is anterodorsally oriented, following the orientation of the adjacent palatine ramus of the pterygoid, with its anterior ramus in the horizontal plane. This differs from *M. carinatus* in which the posterior portion of the palatine is anteroposteriorly oriented and the anterior ramus is in the vertical plane, rising anterodorsally. *Ngwevu* also possesses a laterally extending process of the palatine, but this feature appears to taper to a point and is not bulbous in *Ngwevu*, as it is in *M. carinatus*. This process articulates with the distal portion of the medial surface of the lacrimal. It was thought that the bulbous structure in *M. carinatus* (BP/1/5241) contacted the jugal in life position, but slight disarticulation makes this interpretation ambiguous (Chapelle & Choiniere, 2018).

### Braincase

**Figure 11:**
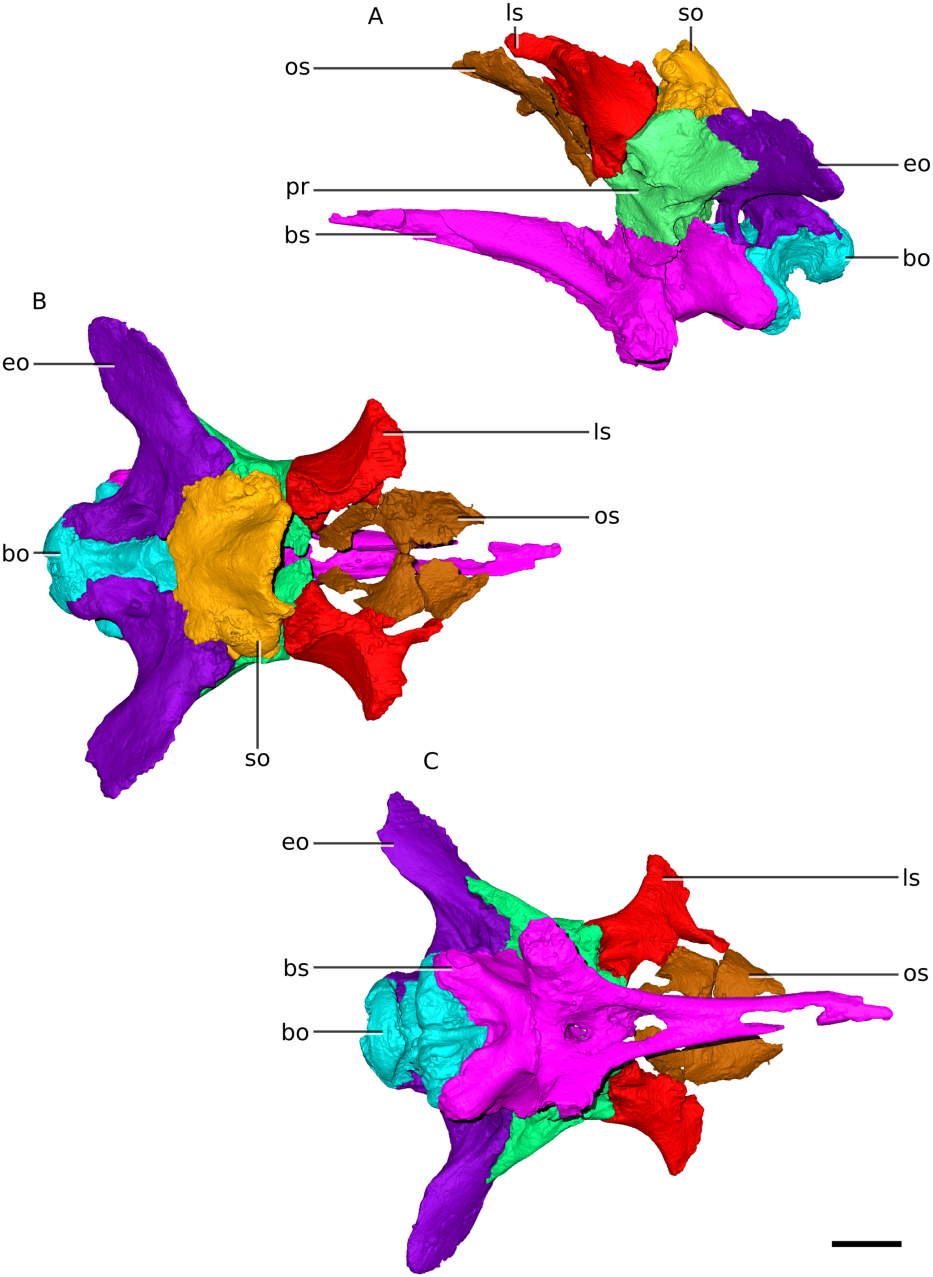
Digital reconstruction of the braincase of BP/1/4779. (A) Left lateral view. (B) Dorsal view. (C) Ventral view. Scale bar represents 10 mm. Abbreviations: bo, basioccipital; bs, basisphenoid; eo, exoccipital; ls, laterosphenoid; os, orbitosphenoid; pr, prootic; so, supraoccipital.

**Figure 12:**
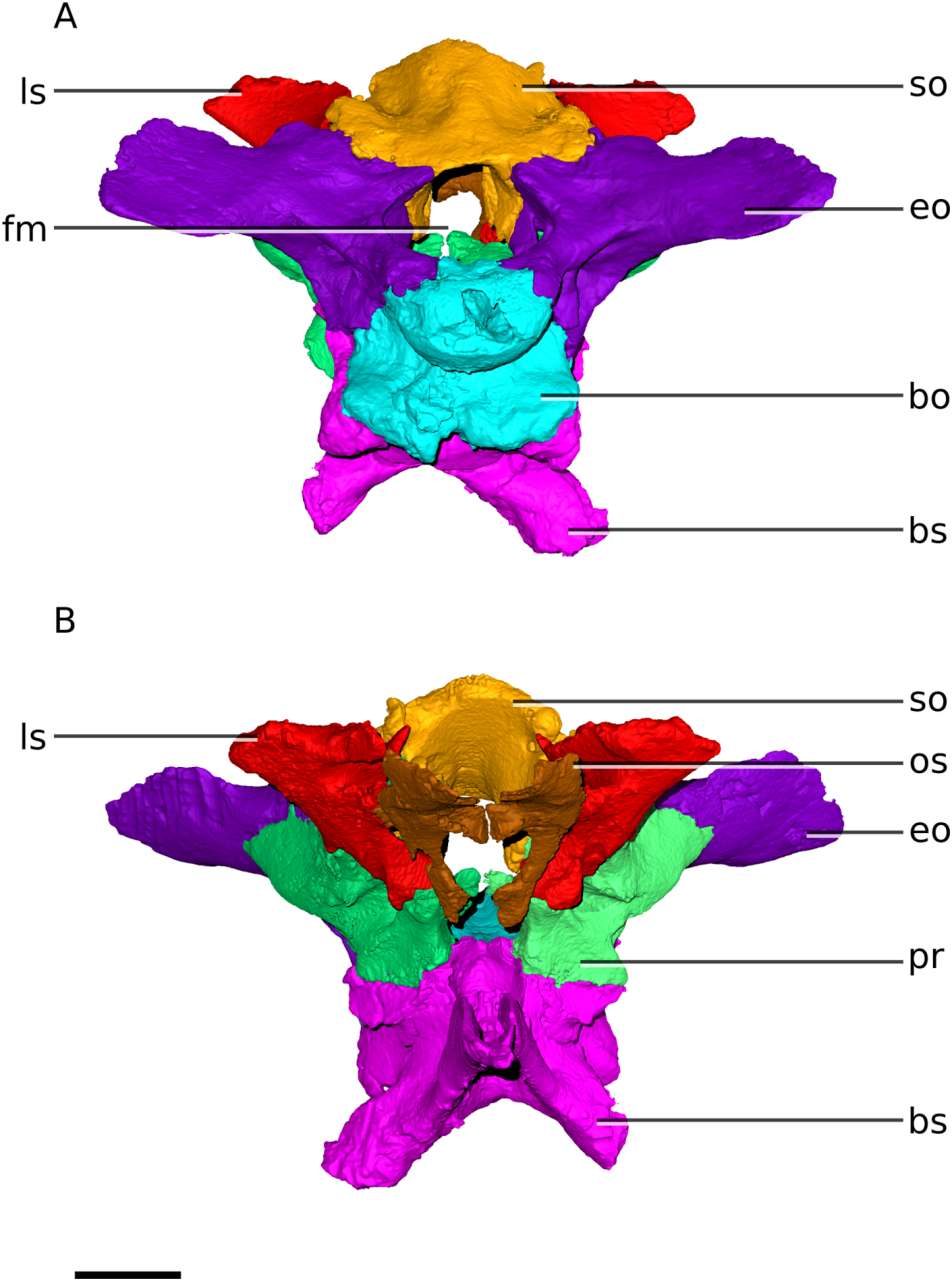
Digital reconstruction of the braincase of BP/1/4779. (A) Posterior view. (B) Anterior view. Scale bar represents 10 mm. Abbreviations: bo, basioccipital; bs, basisphenoid; eo, exoccipital; fm, foramen magnum; ls, laterosphenoid; os, orbitosphenoid; pr, prootic; so, supraoccipital.

The posterior margin of the braincase slopes posteroventrally, probably due to slight distortion of the skull overall (Fig. 11A). All 13 braincase bones are preserved (basisphenoid, prootics, exoccipitals, basioccipital, laterosphenoids, orbitosphenoids, supraoccipital and parietals) (Figs. 11 and 12). Most of the braincase bones have different proportions to *M. carinatus* (Figs. 8 and 9; Table 1). The braincases of BP/1/5241 and BP/1/4376 are also well preserved and used for comparative purposes (Figs. 5C and 5D).

### Orbitosphenoid

The orbitosphenoids of *Ngwevu* have a similar morphology to those of *M. carinatus* (Figs. 5C, 5D and 11A). They are, however, anteroposteriorly shorter and lack the elongated, slender laterosphenoid rami present in *M. carinatus* (main body anteroposterior length at midheight to dorsoventral height ratio of 0.09 in *Ngwevu* and 0.31 in *M. carinatus* adult BP/1/5241).

### Laterosphenoid

**Figure 13:**
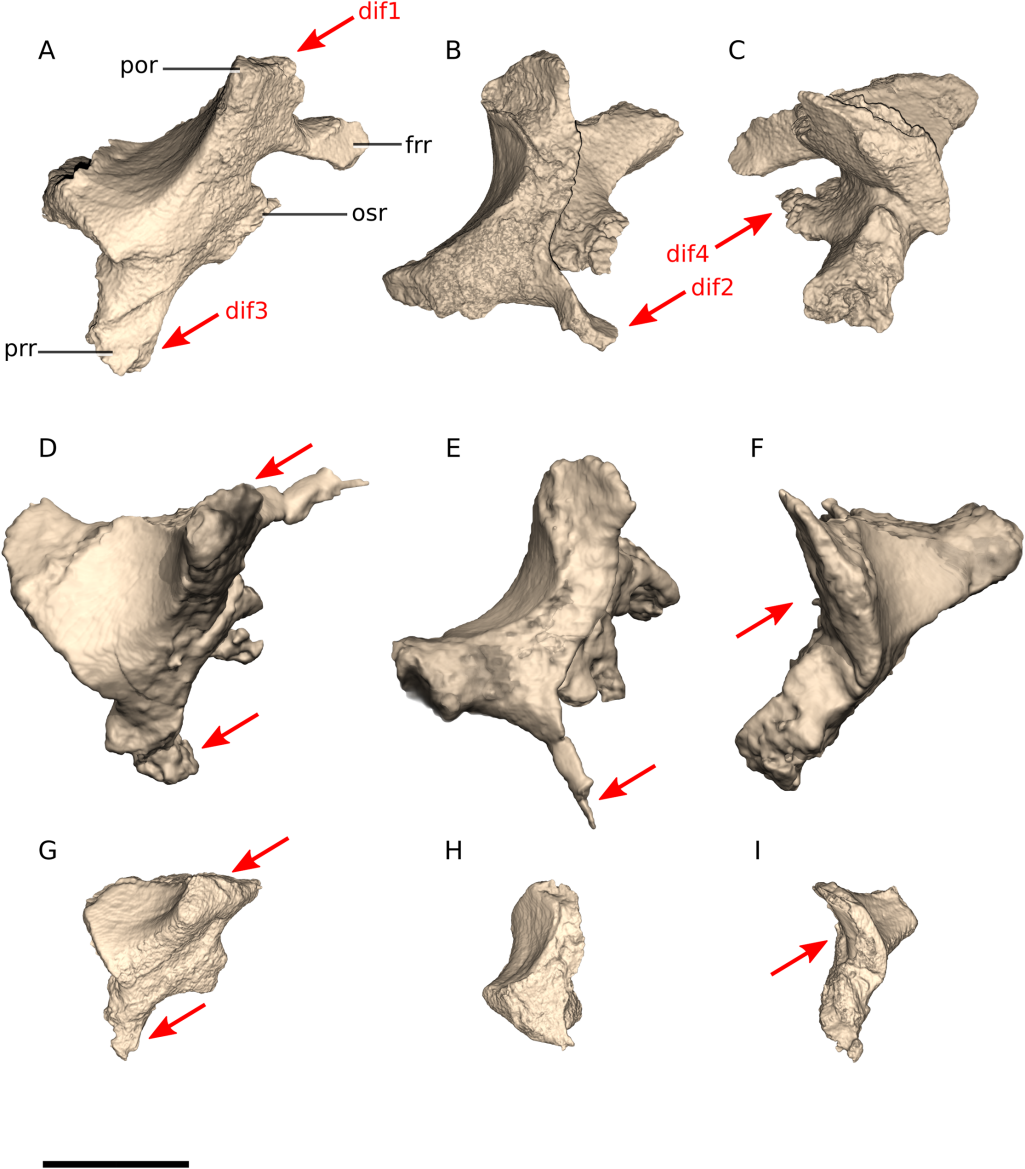
Digital reconstruction of the laterosphenoids of BP/1/4779 and *M. carinatus* adult and juvenile. (A) BP/1/4779 in right lateral view. (B) BP/1/4779 in dorsal view. (C) BP/1/4779 in posterior view. (D) Adult BP/1/5241 in left lateral view. (E) Adult BP/1/5241 in dorsal view. (F) Adult BP/1/5241 in posterior view (BP/1/5241 mirrored to facilitate comparisons). (G) Juvenile BP/1/4376 in right lateral view. (H) Juvenile BP/1/4376 in dorsal view. (I) Juvenile BP/1/4376 in posterior view. Scale bar represents 10 mm. Red arrows point at areas of main differences: dif1, in *Ngwevu*, the postorbital ramus is dorsolaterally oriented, whereas it is laterally oriented in *M. carinatus*; dif2, the frontal ramus of *Ngwevu* is more medially oriented and does not taper to a point as it does in *M. carinatus*; dif3, the prootic ramus of *Ngwevu* extends posteroventrally, as in juvenile *M. carinatus*; dif4, the laterosphenoid of *Ngwevu* is more dorsoventrally compressed than that of *M. carinatus*. Abbreviations: frr, frontal ramus; osr, orbitosphenoid ramus; por, postorbital ramus; prr, prootic ramus.

In *Ngwevu*, the postorbital ramus is dorsolaterally oriented, whereas it is laterally oriented in *M. carinatus* (adults and juvenile) (Fig. 13). The frontal ramus of *Ngwevu* is more medially oriented and does not taper to a point as it does in *M. carinatus*. In *M. carinatus* adults, this ramus is anterodorsomedially oriented. This changes the angle between the frontal ramus and the anterior orbitosphenoid ramus in lateral view: the two rami are separated by a wider angle in *M. carinatus* than in *Ngwevu*, where they are separated by a ‘U’-shaped notch with parallel dorsal and ventral margins (Fig. 13). The prootic ramus of *Ngwevu* extends posteroventrally, as in juvenile *M. carinatus* specimens. In posterior view, the laterosphenoid of *Ngwevu* is more dorsoventrally compressed than that of *M. carinatus* (adult and juvenile), with a more acute angle separating the dorsal and ventral halves of the laterosphenoid (95° in *Ngwevu* and >115° in *M. carinatus* adults and juveniles).

The laterosphenoid in *Coloradisaurus* has a deep, anterodorsally opening notch on its posterior margin that forms the anterior and anterodorsal margins of the trigeminal foramen (CN V). In *Ngwevu* and *M. carinatus*, this posterior margin is gently concave rather than notch-like. The laterosphenoid of *Arcusaurus* also differs from that of *Ngwevu*. The former has a dorsoventrally high and robust anterior orbitosphenoid ramus, a laterally extending postorbital ramus and deep notches along its posterior and anteroventral margins for CN V and CN III, respectively, whereas the anteroventral margin of the laterosphenoid in *Ngwevu* is gently concave rather than notch-shaped.

### Prootic

**Figure 14:**
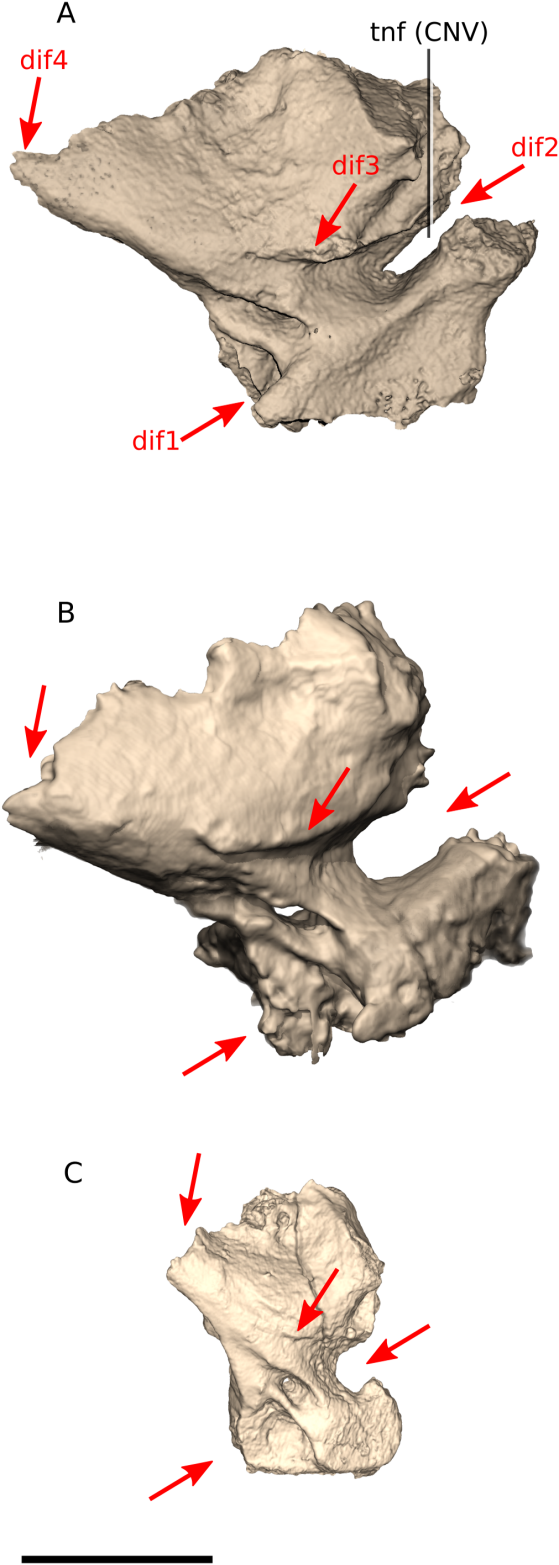
Digital reconstruction of the prootics of BP/1/4779 and *M. carinatus* adult and juvenile. (A) BP/1/4779 in right lateral view. (B) Adult BP/1/5241 in left lateral view (mirrored to facilitate comparisons). (C) Juvenile BP/1/4376 in right lateral view. Scale bar represents 10 mm. Red arrows point at areas of main differences: dif1, in *Ngwevu* and *M. carinatus* adults, the posterior margin of the prootic slopes anteroventrally whereas in juvenile *M. carinatus*, the posterior margin and ventral margin form a near right angle; dif2, in *Ngwevu*, the trigeminal foramen is dorsoventrally compressed compared to that of *M. carinatus*; dif3, the sulcus that extends posteriorly from the dorsal margin of the trigeminal foramen is more prominent in *Ngwevu* and adult *M. carinatus* than in *M. carinatus* juveniles; dif4, the posterodorsal corner of the posterodorsal portion of the prootic is more elongated in *Ngwevu* and forms a more obtuse angle than that of *M. carinatus*. Abbreviations: CNV, cranial nerve V passage; tnf, trigeminal nerve foramen.

Overall, the prootic of *Ngwevu* is similar to that of *M. carinatus* adults, with only small differences (Fig. 14). There do, however, appear to be more prominent ontogenetic differences in *M. carinatus*. In *Ngwevu* and *M. carinatus* adults, the posterior margin of the prootic slopes anteroventrally. In juvenile *M. carinatus*, the posterior margin and ventral margin form a near right angle. This affects the dorsoventral height of the trigeminal nerve foramen (CN V). In juvenile *M. carinatus*, it is relatively large compared those of adults. In *Ngwevu*, however, the trigeminal foramen is very dorsoventrally compressed. The sulcus that extends posteriorly from the dorsal margin of the trigeminal foramen along the lateral surface is more prominent in *Ngwevu* and adult *M. carinatus* than in juveniles of the latter. The posterodorsal corner of the posterodorsal portion of the prootic is more elongated in *Ngwevu* and forms a more obtuse angle (57°) than that of *M. carinatus* adults and juveniles (63° and 85°, respectively). The anteroventral portion of the prootic is also anteroposteriorly shorter in *M. carinatus* juveniles.

The prootic in *Coloradisaurus* appears to have an anteroposteriorly shorter anteroventral portion proportionally when compared to *Ngwevu*, although this is difficult to confirm based on the published figures. It is also more anterodorsally oriented when the basioccipital condyle is held vertically than in *Ngwevu*. This is the opposite of the condition in *M. kaalae* where the prootic is posterodorsally oriented when the basioccipital condyle is held vertically, with the anterior margin of the anteroventral portion being dorsoventrally oriented rather than anterodorsally oriented as in *Ngwevu. M. kaalae* also has an anteroposteriorly short and dorsoventrally elongated prootic (maximum anteroposterior length to dorsoventral height ratio of 0.70 in *M. kaalae*, 0.93 in *Ngwevu*, 0.90 in *M. carinatus* adult BP/1/5241 and 0.85 in *M. carinatus* juvenile).

### Basisphenoid

**Figure 15:**
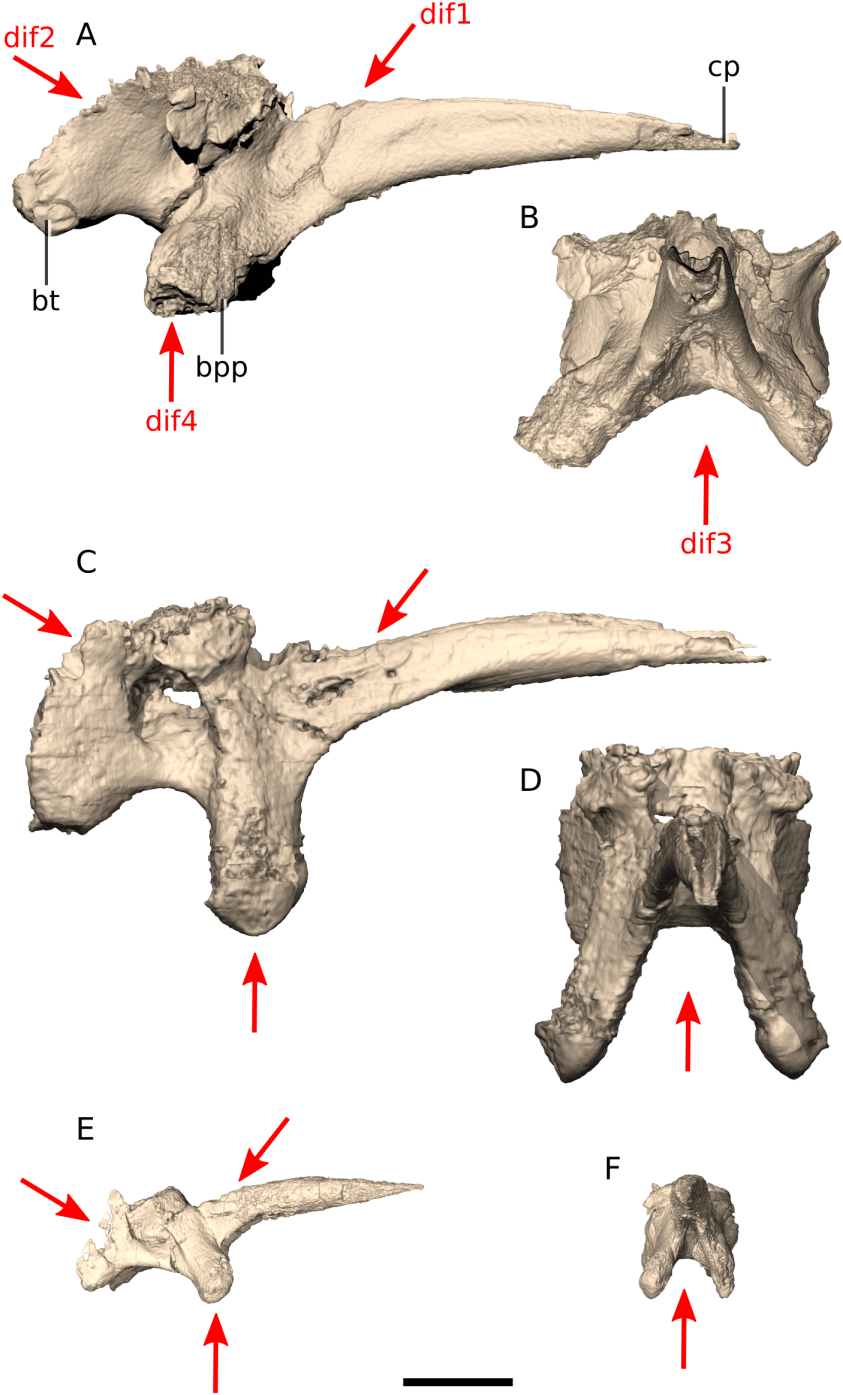
Digital reconstruction of the basisphenoids of BP/1/4779 and *M. carinatus* adult and juvenile. (A) BP/1/4779 in right lateral view. (B) BP/1/4779 in anterior view. (C) Adult BP/1/5241 in right lateral view. (D) Adult BP/1/5241 in anterior view. (E) Juvenile BP/1/4376 in right lateral view. (F) Juvenile BP/1/4376 in anterior view. Scale bar represents 10 mm. Red arrows point at areas of main differences: dif1, *Ngwevu* has a dorsoventrally high cultriform process (at the base) compared to *M. carinatus*; dif2, the posterior margin of the basisphenoid in *Ngwevu* slopes posteroventrally whereas that of *M. carinatus* is dorsoventrally oriented; dif3, in *Ngwevu*, the basipterygoid processes are separated by a wider angle than in *M. carinatus*; dif4, the basipterygoid processes of *Ngwevu* extend slightly posteroventrally whereas those of *M. carinatus* extend ventrally in adults and anteroventrally in juveniles. Abbreviations: bpp, basipterygoid process; bt, basal tuber; cp, cultriform process.

*Ngwevu* has a dorsoventrally compressed basisphenoid main body, but a dorsoventrally high cultriform process (at the base) compared to *M. carinatus* (Fig. 15), with a dorsoventral height of the base to anteroposterior length of the process ratio of 0.27 in *Ngwevu* and 0.22 and 0.19 in *M. carinatus* adults and juveniles, respectively. It is also proportionally mediolaterally wider at its base than in *M. carinatus* adults (cultriform process base mediolateral width to anteroposterior length ratio of 0.26 in *Ngwevu* and 0.21 and 0.13 in *M. carinatus* adults and juveniles, respectively). The posterior margin of the basisphenoid in *Ngwevu* slopes posteroventrally whereas that of *M. carinatus* (adults and juvenile) is dorsoventrally oriented. The posteroventral sloping seen in BP/1/4779 may be due to slight distortion of the skull. In *Ngwevu*, the basipterygoid processes are separated by 91° in posterior/anterior view whereas those of *M. carinatus* adults are separated by a 35° or 60° angle (BP/1/5241 and BP/1/4934, respectively) and 45° in the juvenile. In *Adeopapposaurus*, *Sarahsaurus* and *Lufengosaurus huenei* this angle is more similar to that of *Ngwevu* (>60°), whereas in *Coloradisaurus* and *M. kaalae* the state is more similar to *M. carinatus* (<60°). The basipterygoid processes of *Ngwevu* extend slightly posteroventrally in lateral view whereas those of *M. carinatus* adults extend ventrally. In juvenile *M. carinatus*, the processes extend anteroventrally. In *Adeopapposaurus* and *Lufengosaurus huenei* these processes extend ventrally, in *Coloradisaurus* and *M. kaalae* they extend slightly posteroventrally and in *Sarahsaurus* they extend anteroventrally.

The basal tubera of *Ngwevu* and *M. carinatus* adults do not extend as far ventrally as the basipterygoid processes whereas in *M. carinatus* juveniles, these extend to approximately the same level. The basal tubera of *Ngwevu* are separated by 90° in dorsal/ventral view whereas in *M. carinatus*, this angle is approximately 40° (in both adults and juveniles). BP/1/4376 appears to be slightly mediolaterally compressed, which may be exaggerating the acuteness of the angles between the basal tubera and basipterygoid processes.

### Exoccipital/opisthotics (‘otoccipitals’)

**Figure 16:**
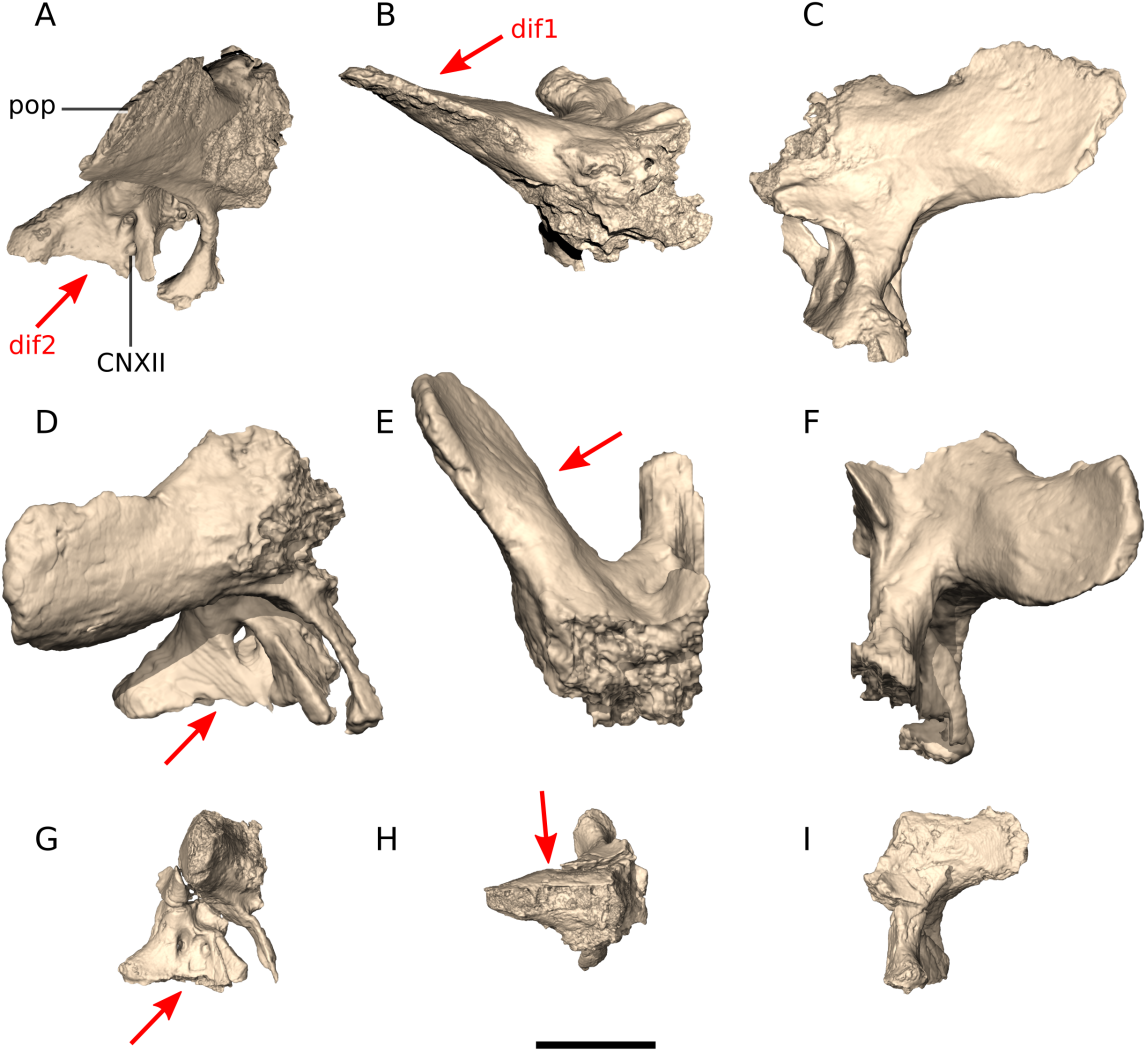
Digital reconstruction of the exoccipitals of BP/1/4779 and *M. carinatus* adult and juvenile. (A) BP/1/4779 in right lateral view. (B) BP/1/4779 in dorsal view. (C) BP/1/4779 in posterior view. (D) Adult BP/1/5241 in right lateral view. (E) Adult BP/1/5241 in dorsal view. (F) Adult BP/1/5241 in posterior view. (G) Juvenile BP/1/4376 in right lateral view. (H) Juvenile BP/1/4376 in dorsal view. (I) Juvenile BP/1/4376 in posterior view. Scale bar represents 10 mm. Red arrows point at areas of main differences: dif1, in *Ngwevu*, the paroccipital processes form a wider angle than in *M. carinatus* adults (difficult to assess in *M. carinatus* juveniles due to preservation); dif2, the ventral margin of the main body of the otoccipital slopes anteroventrally in *Ngwevu* whereas in *M. carinatus* it is anteroposteriorly oriented. Abbreviations: CNXII, cranial nerve XII passage; pop, paroccipital process.

In *Ngwevu*, the exoccipitals are fused to the opisthotics, as in most adult dinosaurs. The otoccipital displays a lot of variation within the sample (Fig. 16). One of the major differences between the otoccipitals of *Ngwevu* and adult *M. carinatus* is the angle separating the paroccipital processes. In *Ngwevu*, these processes form an angle of approximately 130° whereas in adult *M. carinatus* this angle is either 67° (BP/1/5241) or 105° (BP/1/4934). In the juvenile this angle is 120°, but this region of the skull is poorly preserved. Other massospondylids have angles separating the paroccipital processes that are <110° (90° in *Sarahsaurus*, 70° in *Lufengosaurus huenei*, 105° in *Coloradisaurus* and 97° in *Adeopapposaurus*). The ventral margin of the main body of the otoccipital slopes anteroventrally in *Ngwevu* whereas in *M. carinatus* it is anteroposteriorly oriented (in both adults and juvenile).

The hypoglossal cranial nerve (CN XII) and the positions of the openings for the associated branches of this nerve vary between all four specimens. *Ngwevu* has two foramina in lateral view with one being dorsal to the other and two foramina in medial view but positioned next to each other with one being anterior to the other. BP/1/4376 has two foramina both laterally and medially, with one foramen positioned anteriorly to the other. BP/1/5241 has one foramen in lateral view and two in medial view, positioned next to each other with one being anterior to the other. Finally, BP/1/4934 only has one foramen in lateral and in medial view. In Archosauria, the positions of the hypoglossal nerve openings are known to vary interspecifically, intraspecifically and even within one specimen with the contralateral sides having different foramina arrangements (Mayr, 2018).

### Basioccipital

The basioccipital of *Ngwevu* does not differ from that of *M. carinatus* in any significant features (Figs. 1, 2 and 5).

### Supraoccipitial

In *Ngwevu*, the supraoccipital is mediolaterally wider than it is dorsoventrally high (Figs. 2B and 4B) whereas in *M. carinatus*, the supraoccipital is almost as wide as it is high (supraoccipital mediolateral width to dorsoventral height ratio of 1.44 in *Ngwevu*, 0.94 in BP/1/4934 and 0.99 in BP/1/5241). The supraoccipital does not differ from that of *M. carinatus* in any other significant features.

### Parietal

The parietals are fused in BP/1/4779 (Figs. 1B and 3B). The squamosal rami of the parietals contact the paroccipital processes and therefore also diverge from each other at an angle of approximately 130°. This differs from the condition in *M. carinatus* adults where the angle is 65–67°. In the juvenile this angle is 120°, but this region of the skull is poorly preserved. Other massospondylids have angles similar to those separating the paroccipital processes (see above). In *Ngwevu*, the parietal is proportionally broad and has a maximum anteroposterior length to mediolateral width of the body ratio of 1.4. In *M. carinatus* adults, this ratio exceeds 2.0, but in the juvenile this ratio is close to 1.0, although this region of the skull is poorly preserved.

### Mandible

**Figure 17:**
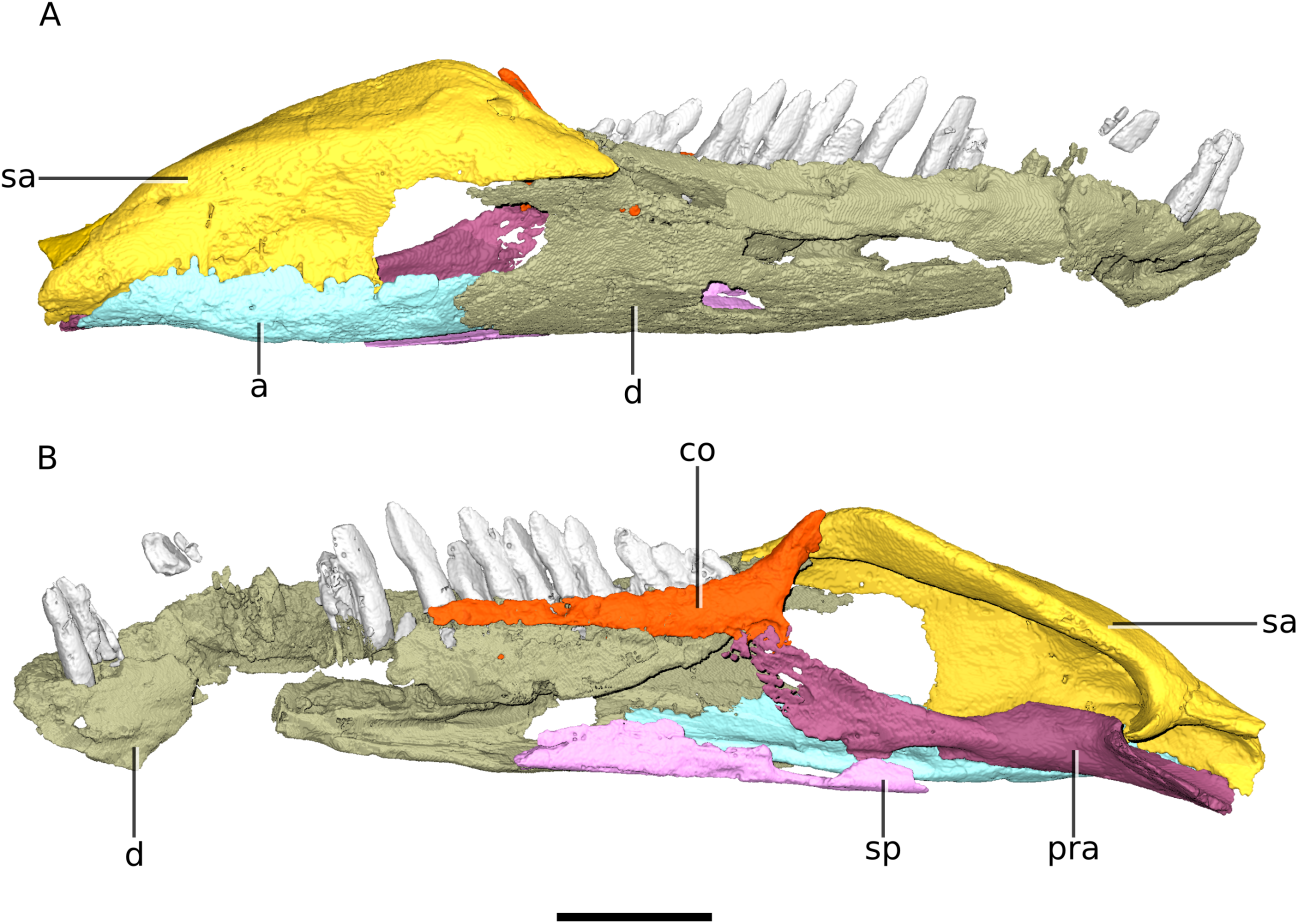
Digital reconstruction of the right mandible of BP/1/4779. (A) Lateral view. (B) Medial view. Scale bar represents 10 mm. Abbreviations: a, angular; co, coronoid; d, dentary; pra, prearticular; sa, surangular; sp, splenial.

Two features of the mandible help to distinguish *Ngwevu* from other massospondylids (Fig. 17). Firstly, the anterior margin of the dentary symphysis is linear and slopes strongly posteroventrally, although this might have been accentuated by breakage. This is similar to the morphology in *M. kaalae*, *Arcusaurus* and *Adeopapposaurus*. Juvenile *M. carinatus* (BP/1/4376), *Sarahsaurus* and *Coloradisaurus* have a dorsoventrally oriented and convex anterior dentary margin. An adult *M. carinatus* (BP/1/4934) has a more strongly convex anterior margin although it is slightly posteroventrally sloping. The latter also exhibits dorsoventral expansion of the anterior portion of the dentary, with the anterior portion of the ventral margin sloping anteroventrally.

Secondly, the coronoid region of the mandible is proportionally high in *Ngwevu*. This is associated with a steep anteroventrally sloping anterior margin of the coronoid eminence. In *M. carinatus* (BP/1/4934 and BP/1/4376), *Coloradisaurus* and *Adeopapposaurus* the anterior margin of the coronoid region slopes more gently anteroventrally. In *M. kaalae* this margin is also steep, however the coronoid region is not as dorsoventrally high.

### Dentition

*Ngwevu* has four premaxillary teeth, a minimum of 18 maxillary teeth and a minimum of 21 dentary teeth (Figs. 1, 3 and 17). *M. carinatus* has four premaxillary teeth, 14–22 maxillary teeth (BP/1/4376, BP/1/5241 and BP/1/4934 have 14, 17 and 22 maxillary teeth, respectively) and 15–26 dentary teeth (15 and 26 teeth in BP/1/4376 and BP/1/4934, respectively). *M. kaalae* has four premaxillary teeth, 15–16 maxillary teeth and 18–19 dentary teeth. *Sarahsaurus aurifontanalis* has four premaxillary teeth, 16 maxillary teeth and 20 dentary teeth. *Lufengosaurus huenei* has 20 maxillary teeth and a minimum of 16 dentary teeth; *Coloradisaurus* has 22 maxillary teeth and a minimum of 18 dentary teeth.

The dental morphology of *Ngwevu* is similar to that of other massospondylids with a semi-spatulate shape, coarse serrations/denticles on the apical third of the tooth crown, and imbricating with the distal side of the tooth overlapping the mesial side of the succeeding tooth. Although not visible on the digital reconstructions due to lack of resolution, the specimen displays apicobasal fluting on the labial surface of the teeth (Fig. 2C). This is seen in *M. carinatus* (BP/1/5241 and BP/1/4934), *M. kaalae*, *Pulanesaura eocollum* and *Coloradisaurus*, though the fluting present in *Ngwevu* is not as pronounced as in *Pulanesaura* (McPhee et al., 2015).

### Postcranial anatomy

**Figure 18:**
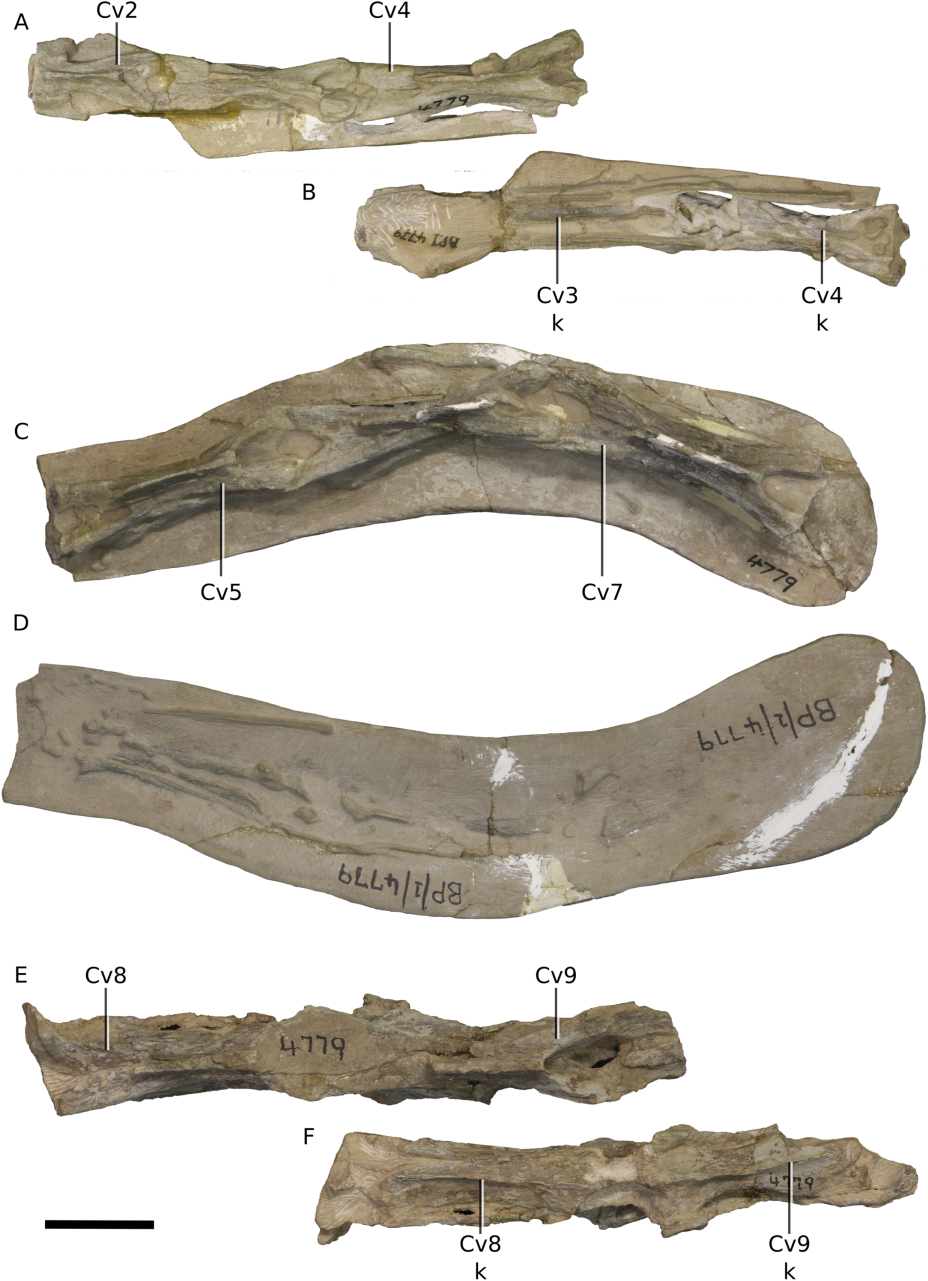
Photographs of the cervical vertebrae of BP/1/4779. (A) Block 2, cervical vertebrae 2–4 in dorsal view. (B) Block 2, cervical vertebrae 2–4 in ventral view. (C) Block 3, cervical vertebrae 5–7 in dorsal view. (D) Block 3, cervical vertebrae 5–7 in ventral view. (E) Block 4, cervical vertebrae 8–9 in dorsal view. (F) Block 4, cervical vertebrae 8–9 in ventral view. Scale bar represents 50 mm. Abbreviations: Cv, cervical vertebra; k, keel. Photographs by Kimberley E.J. Chapelle.

**Figure 19:**
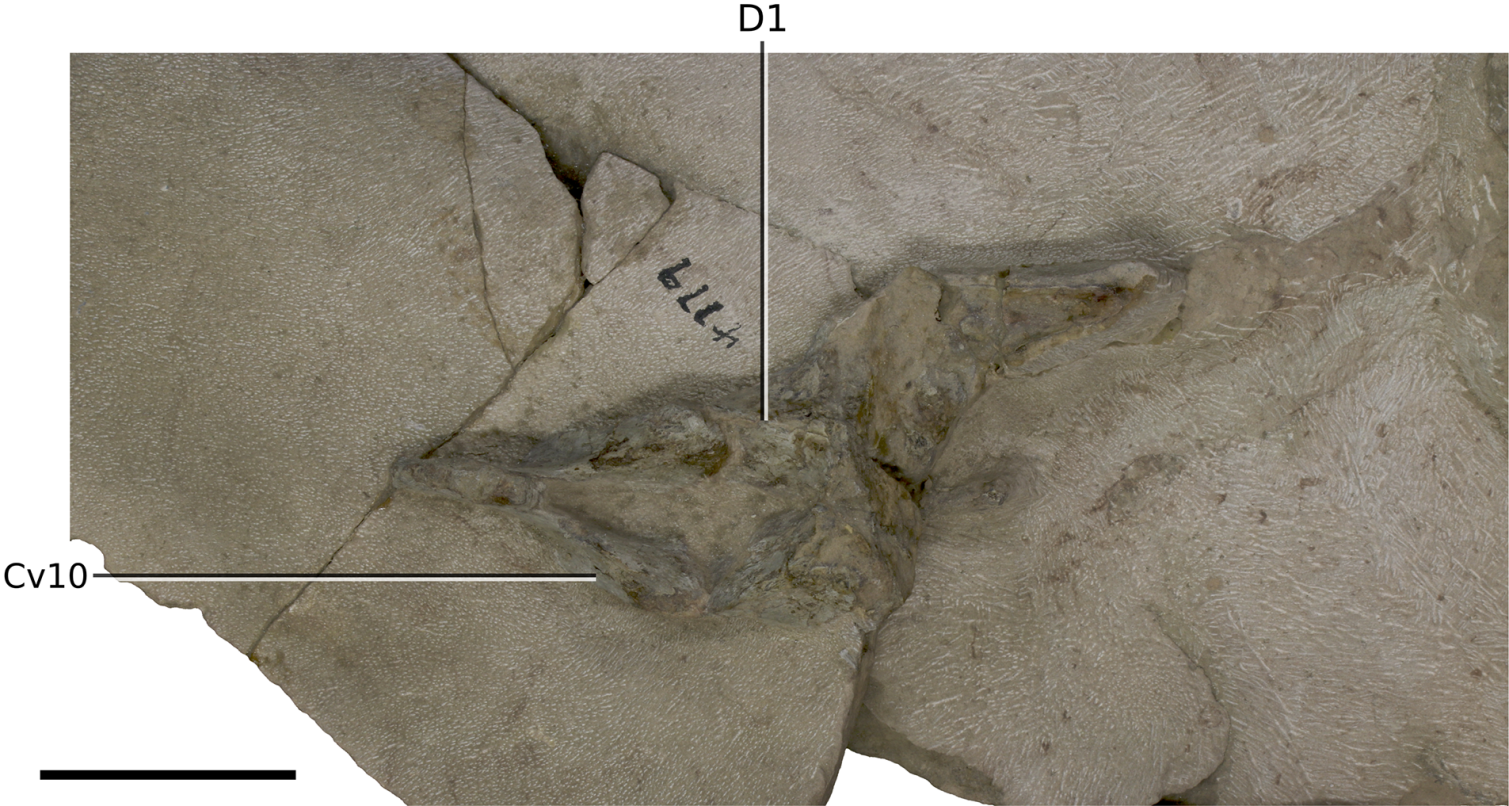
Photograph of block 5 containing the last cervical vertebra and the anterior portion of the first dorsal vertebra. Scale bar represents 50 mm. Abbreviations: Cv, cervical vertebra; D, dorsal vertebra. Photograph by Kimberley E.J. Chapelle.

**Figure 20:**
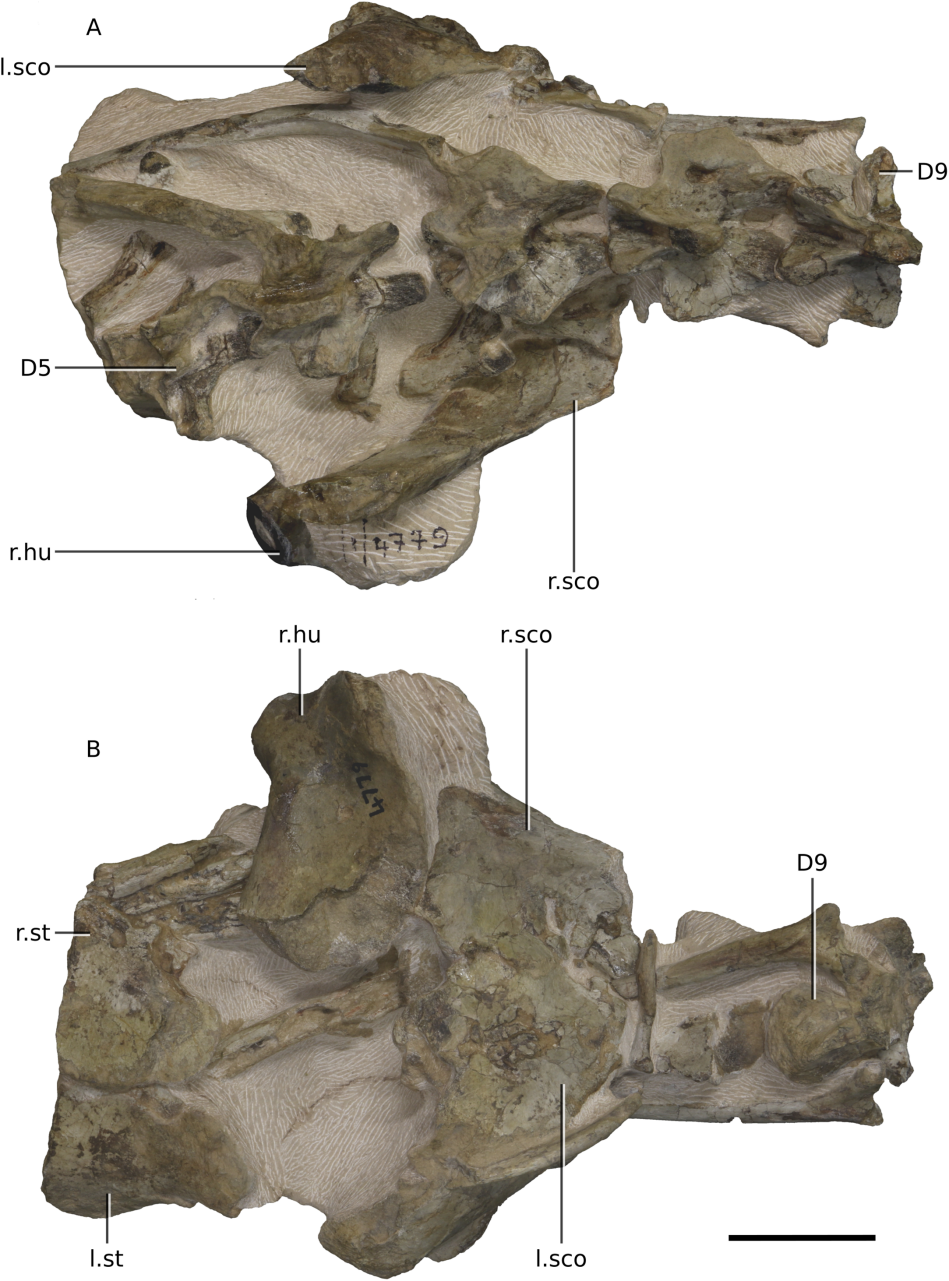
Photographs of block 6 containing the anterior thorax. (A) Dorsal view. (B) Ventral view. Scale bar represents 50 mm. Abbreviations: D, dorsal vertebra; hu, humerus; l., left; r., right; sco, scapula-coracoid; st, sternal plate. Photographs by Kimberley E.J. Chapelle.

**Figure 21:**
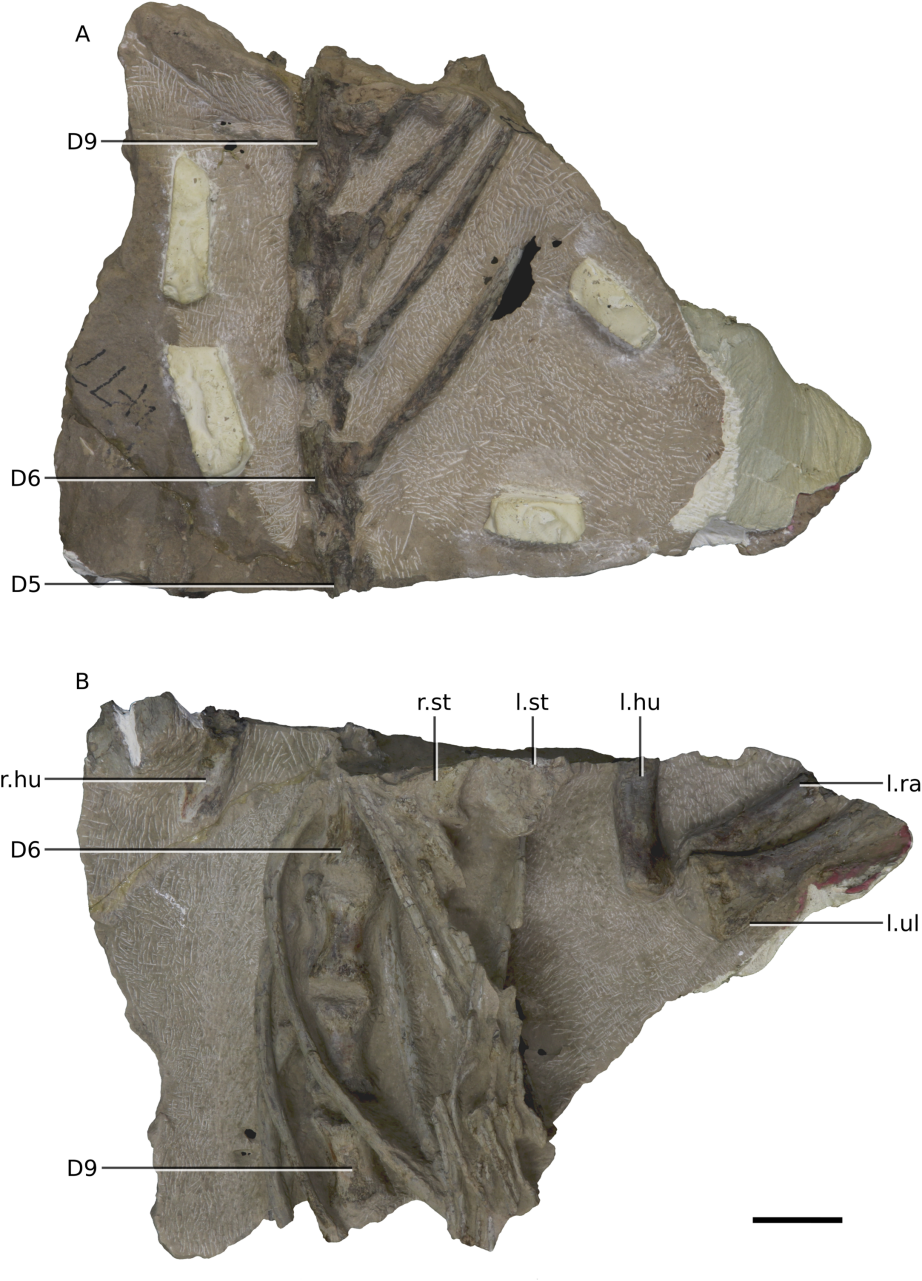
Photographs of block 7 containing the posterior thorax. (A) Dorsal view. (B) Ventral view. Scale bar represents 50 mm. Abbreviations: D, dorsal vertebra; hu, humerus; l., left; r., right; ra, radius; st, sternal plate; ul, ulna. Photographs by Kimberley E.J. Chapelle.

**Figure 22:**
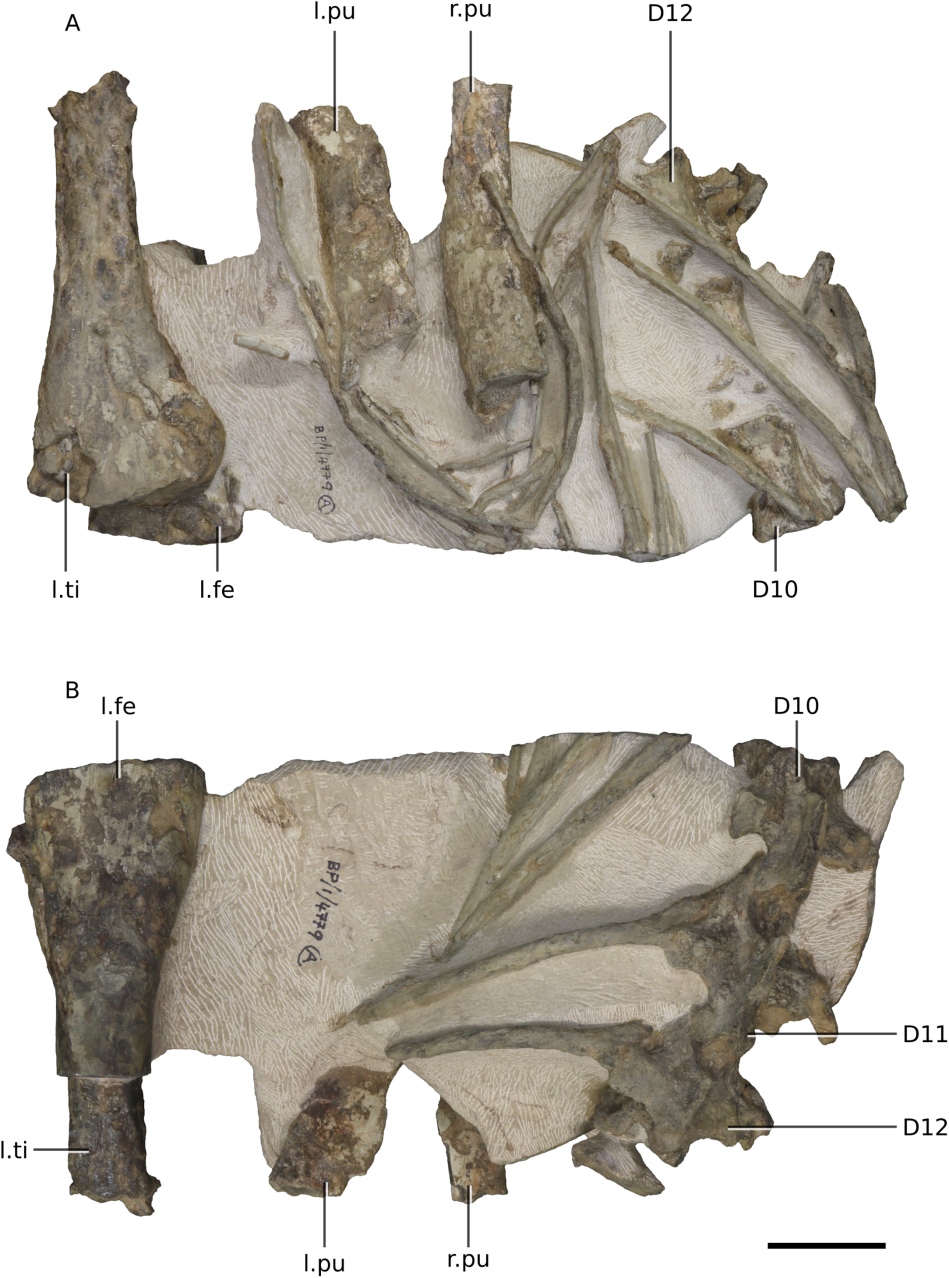
Photographs of block 8 containing portions of the pelvic girdle. (A) Dorsal view. (B) Ventral view. Scale bar represents 50 mm. Abbreviations: D, dorsal vertebra; fe, femur; l., left; pu, pubis; r., right; ti, tibia. Photographs by Kimberley E.J. Chapelle.

**Figure 23:**
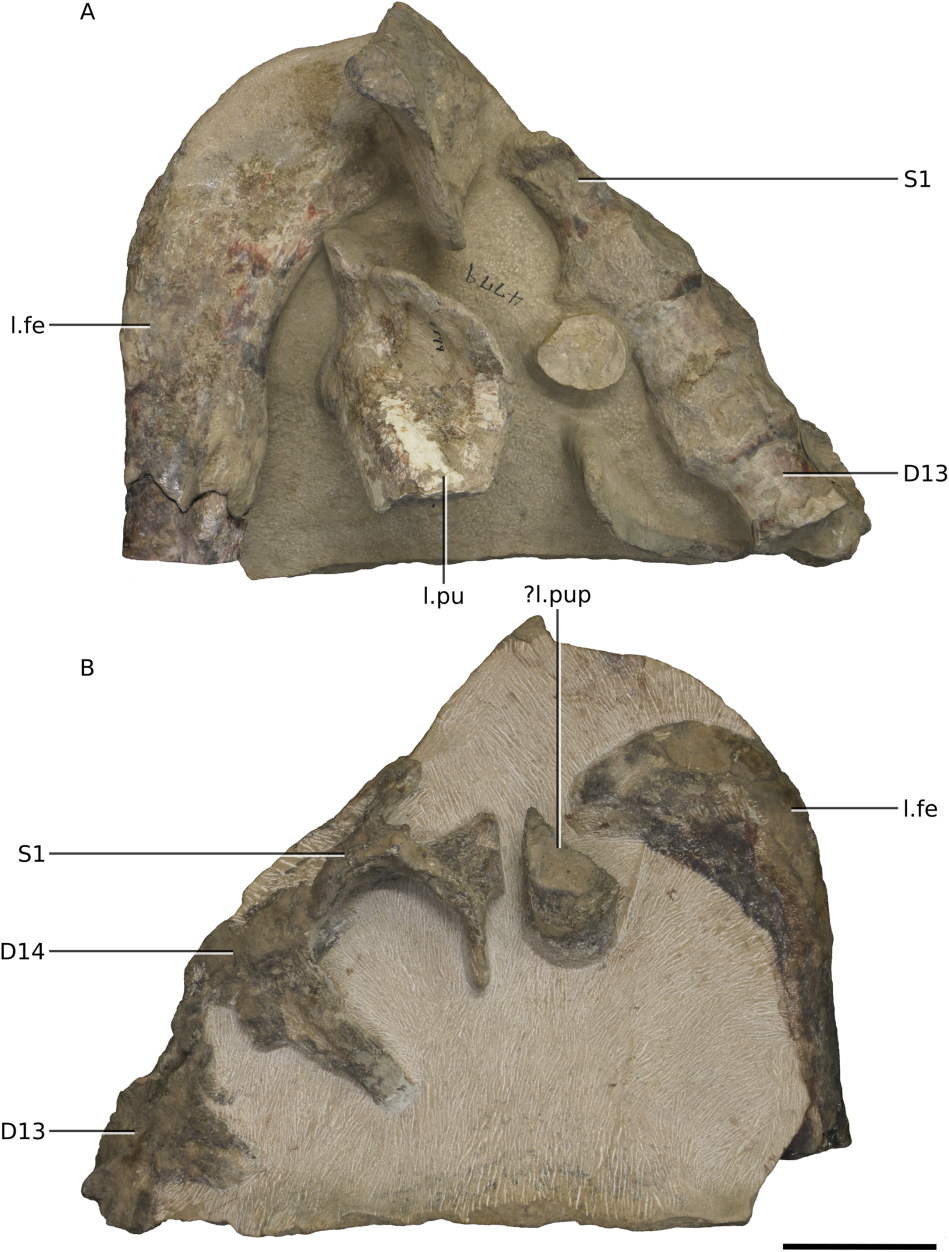
Photographs of block 9 containing portions of the sacrum. (A) Dorsal view. (B) Ventral view. Scale bar represents 50 mm. Abbreviations: D, dorsal vertebra; fe, femur; l., left; pu, pubis; pup, pubic peduncle of ilium; S, sacral vertebra. Photographs by Kimberley E.J. Chapelle.

**Figure 24:**
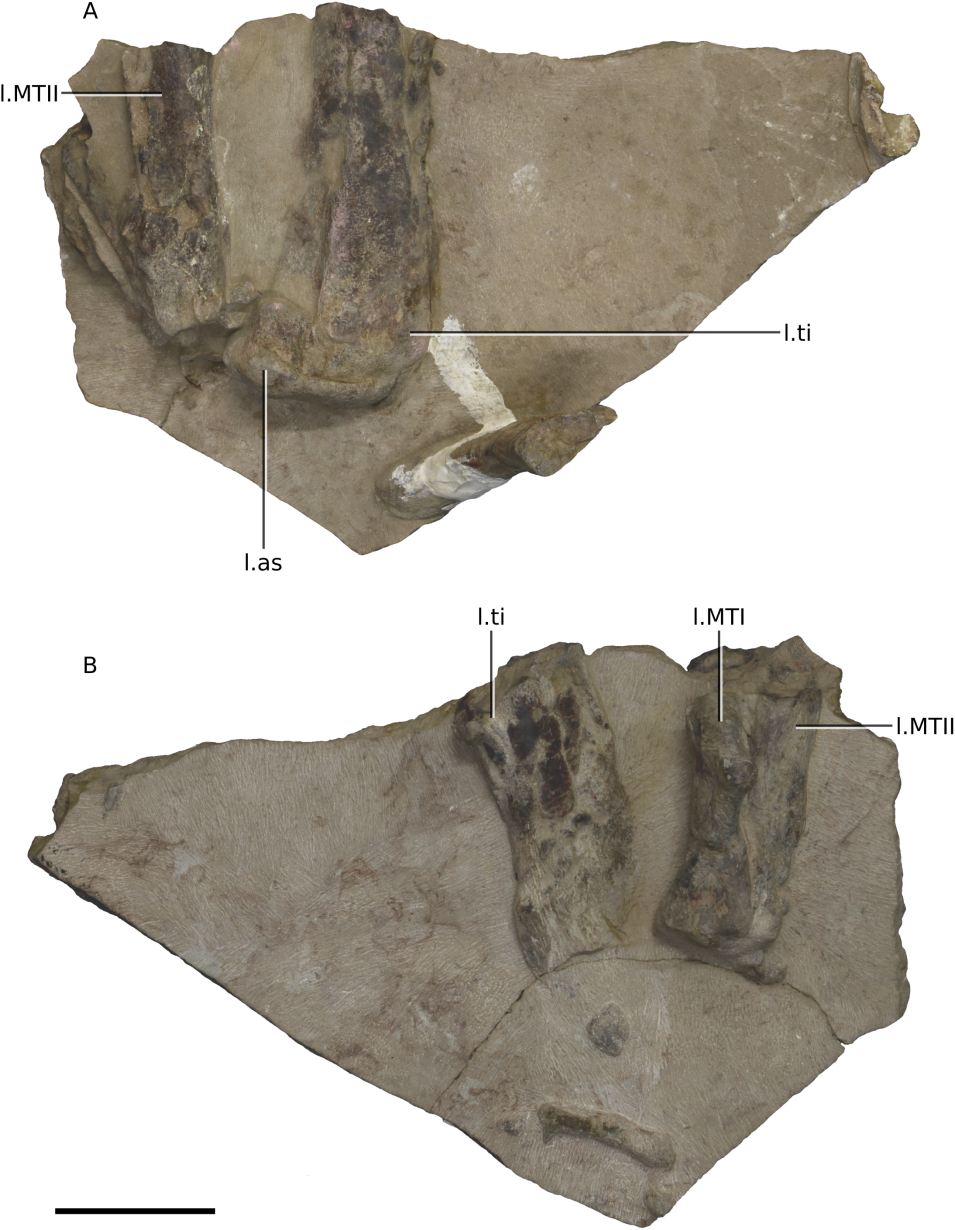
Photographs of block 10 containing a partial left ankle. (A) Posterior view. (B) Anterior view. Scale bar represents 50 mm. Abbreviations: as, astragulus; l., left; MT, metatarsal; ti, tibia. Photographs by Kimberley E.J. Chapelle.

**Figure 25:**
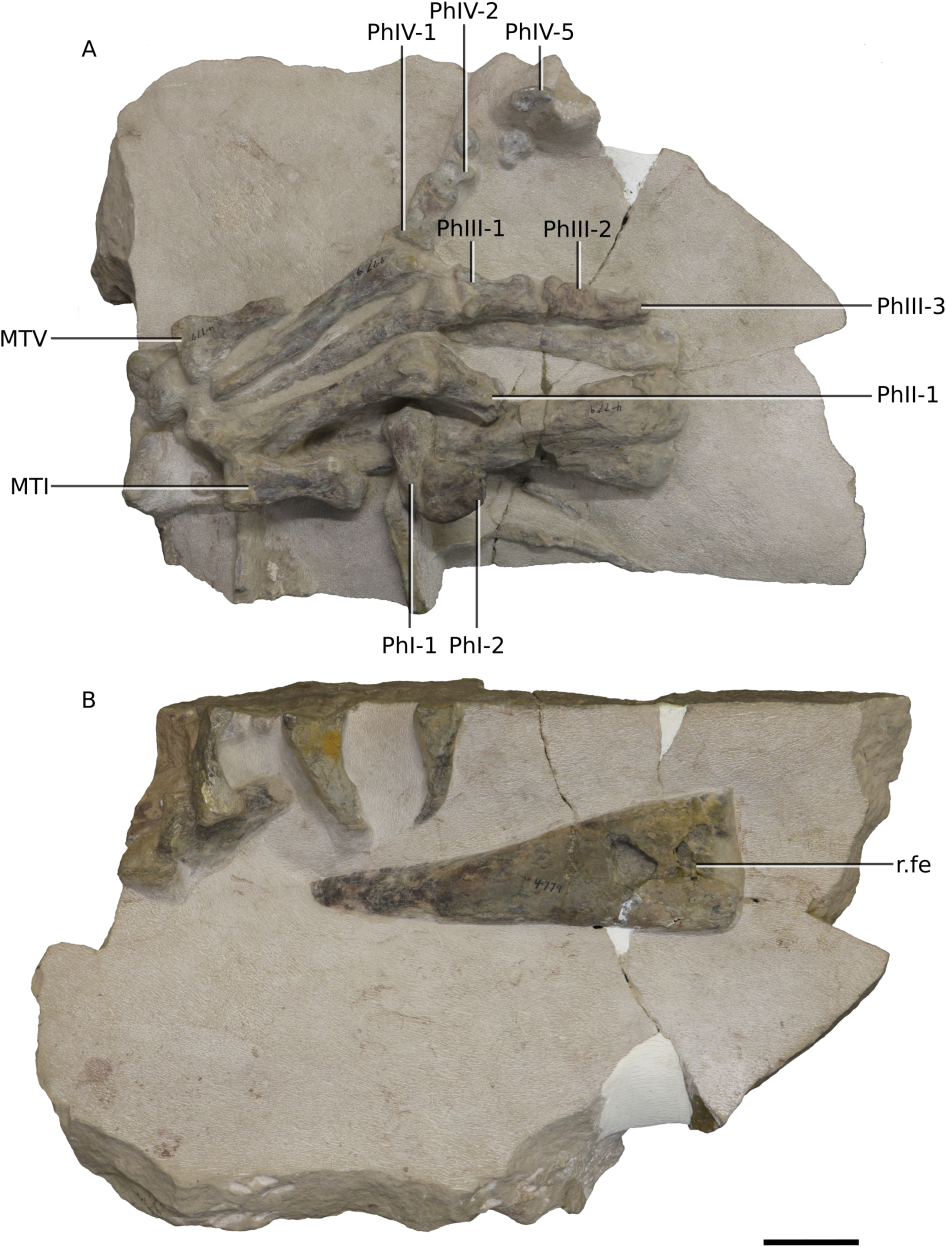
Photographs of block 11 containing a complete right foot and femur. (A) Dorsal view. (B) Ventral view. Scale bar represents 50 mm. Abbreviations: fe, femur; MT, metatarsal; Ph, phalanx; r., right. Photographs by Kimberley E.J. Chapelle.

**Figure 26:**
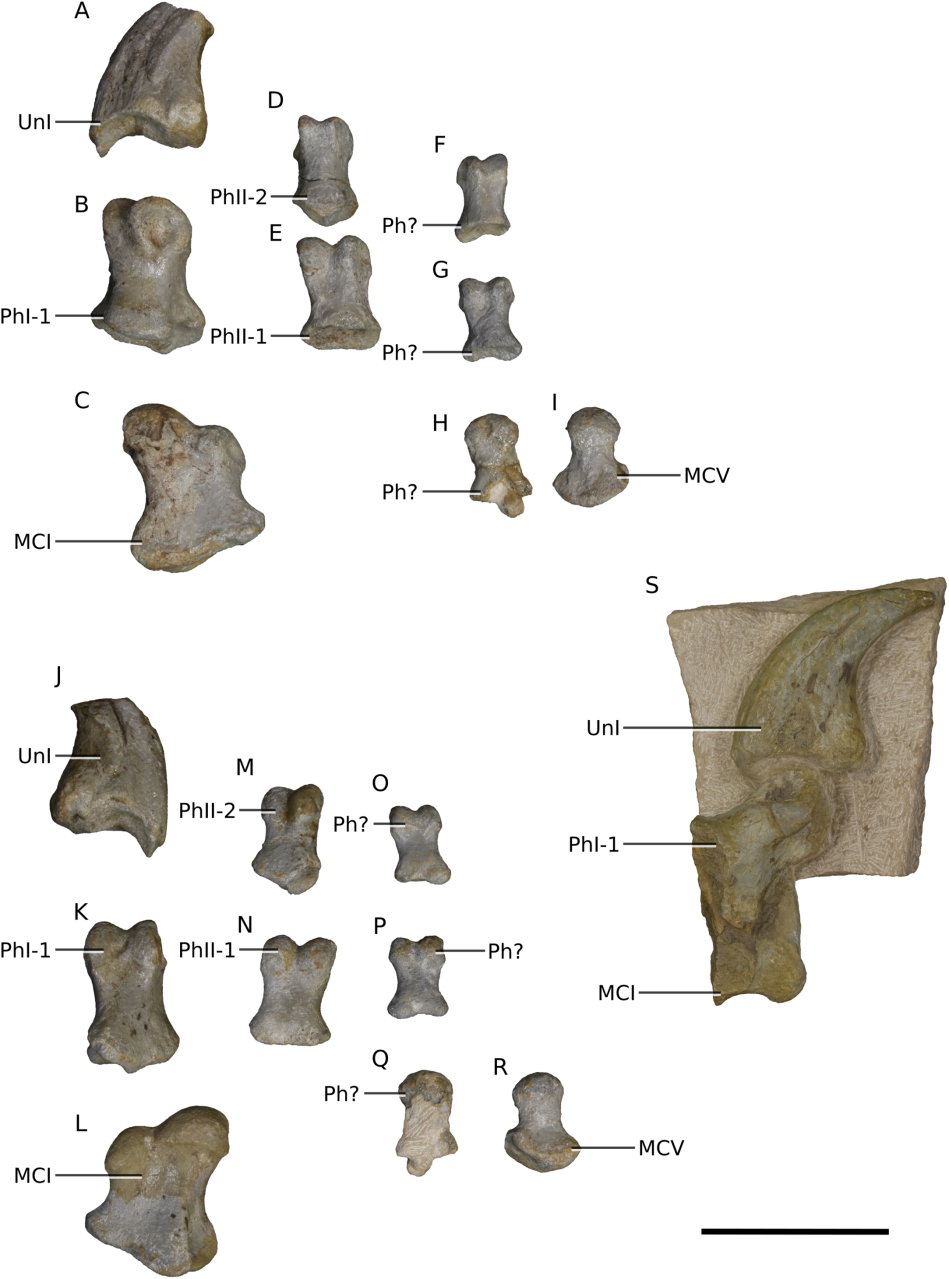
Photographs of elements pertaining to the manus. (A) Left ungual I in medial view. (B) Left phalanx I-1 in dorsal view. (C) Left metacarpal I in dorsal view. (D) Left phalanx II-2 in dorsal view. (E) Left phalanx II-1 in dorsal view. (F–H) Unidentified phalanges in dorsal view. (I) Left metacarpal V in dorsal view. (J) Left ungual I in lateral view. (K) Left phalanx I-1 in ventral view. (L) Left metacarpal I in ventral view. (M) Left phalanx II-2 in ventral view. (N) Left phalanx II-1 in ventral view. (O–Q) Unidentified phalanges in ventral view. (R) Left metacarpal V in ventral view. (S) Right partial manual digit one in lateral view. Scale bar represents 50 mm. Abbreviations: MC, metacarpal; Ph, phalanx; Un, ungual. Photographs by Kimberley E.J. Chapelle.

BP/1/4779 also includes a near complete and almost fully articulated postcranial skeleton, which has been prepared so that it is currently preserved in relief in 11 separate blocks. These blocks contain the following material: block (1) complete articulated skull (see above) and articulated atlas; block (2) axis and cervical vertebrae (Cv) 3 and 4; block (3) Cv5–7; block (4) Cv8, Cv9 and anterior part of 10; block (5) posterior part of cervical 10 and anterior part of dorsal 1; block (6) posterior part of dorsal 1, dorsals 2–4, anterior dorsal 5, left and right coracoids and proximal scapulae, ribs, anterior portion of left and right sternal plates, proximal right humerus; block (7) posterior half of left and right sternal plates, distal left and right humeri, proximal left ulna and radius, posterior half of dorsal 5, dorsal vertebrae 6–9; block (8) dorsal vertebrae 10–12, distal left and right pubes, distal left femur, proximal left tibia; block (9) proximal left femur, proximal left pubis, dorsal vertebrae 13 and 14, primordial sacral vertebra 1, a possible pubic peduncle of the left ilium; block (10) distal left tibia, left astragalus, a complete left metatarsal I and partial metatarsal II, a possible ischial fragment; and block (11) the complete right femur and almost complete right foot (lacking some unguals) (Figs. 18–26).

The postcranial skeleton is visible mostly in dorsal and ventral views. Although very complete, the cortical surfaces of the bones are poorly preserved, as are the proximal and distal ends of the limb bones, rendering it difficult to describe or view potential distinguishing features. Due to the current state of preparation and preservation of the bones, very little can be said regarding the detailed anatomy of the dorsal vertebrae, long bones, shoulder and pelvic girdle elements (Figs. 20–25). Measurements are also hindered by the presence of matrix.

### Vertebral column

BP/1/4779 has a fully articulated vertebral series that comprises 10 Cv (Figs. 18 and 19), 14 dorsal vertebrae and one primordial sacral vertebra (Figs. 19–23). There is no distinction between the transverse process shape or orientation of D13 and the vertebra between D13 and the first primordial sacral (Fig. 23), but a dorsal rib articulates with the transverse process of this vertebra. For this reason, we consider it to be the last dorsal vertebra (D14) and not a dorsosacral. The neural arches are tightly fused to the centra suggesting that BP/1/4779 was nearly fully grown. The lack of a dorsosacral vertebra is a potential key distinction from *M. carinatus*, which has 10 Cv, 14 dorsal vertebrae, one dorsosacral and two primordial sacral vertebrae. *Adeopapposaurus* possesses 11 Cv, 13 dorsal vertebrae, one dorsosacral and two primordial sacrals. *Sarahsaurus* and *Lufengosaurus huenei* have the same vertebral formula as *M. carinatus* overall (although it is unclear if a dorsosacral or caudosacral has been incorporated as the third sacral vertebra in *Lufengosaurus huenei*). *Ignavusaurus* possesses 14 dorsal vertebrae, one dorsosacral vertebra and two sacral vertebrae.

The Cv of BP/1/4779 are undistorted (Figs. 18 and 19). Only Cv4, Cv8 and Cv9 are visible in lateral and ventral views, the others remaining encased in matrix. Cv4 and Cv8 possess a distinct ‘stepped’ morphology between their neural arch and centra, with the neural arch pedicles overhanging the dorsolateral surface of the centrum as in *M. carinatus*, *Leyesaurus* and *Adeopapposaurus* (Barrett et al., 2019). In Cv4, the centrum is exposed and has an anteroposterior length to dorsoventral height ratio of approximately 7.15. This is almost as elongate as in *M. carinatus* where the ratio is between 7.5 and 7.6 for Cv3 and Cv4, respectively (Barrett et al., 2019). This is, however, much more elongate than those of *Adeopapposaurus* (ratio of 5.0), *Coloradisaurus* (ratio of 3.8), *Lufengosaurus huenei* (ratio of 3.3) and *Leyesaurus* (ratio of 5.0). A distinct ventral keel that extends along the midline of the ventral centrum surface is visible in all cervicals but Cv7.

### Manus

A few isolated manual elements are preserved in BP/1/4779 including the left metacarpal (MC) I, phalanges I-1 and I-2 (ungual), phalanges II-1 and II-2, MC V, three unidentified phalanges, and a right partial articulated first digit (partial MC I, complete phalanges I-1 and I-2) (Fig. 26).

The proportions of the first metacarpal differ between BP/1/4779 and *M. carinatus* (BP/1/4934 and BP/1/4376). The proximal mediolateral width to proximodistal length ratio is of 0.74 in *Ngwevu* whereas it is close to 1.0 in *M. carinatus. Adeopapposaurus* has similar proportions to *M. carinatus* with a ratio of 0.83 whereas *Sarahsaurus* has more gracile proportions, as in *Ngwevu*, with a ratio of 0.71.

The first phalanx of the first digit also appears to be proportionally more gracile in *Ngwevu* than in *M. carinatus*. The latter has a robust protuberance on the plantar surface of the proximal end, with a proximal dorsoventral height to proximodistal length ratio of 0.92. *Ngwevu* does not have this accentuated feature and has a ratio of 0.58. This is difficult to compare in other massospondylids.

### Pes

BP/1/4779 has an almost complete right foot lacking only phalanges from digit II and unguals II and III. There are no notable differences between the pes of *Ngwevu* and that of *M. carinatus* (Fig. 25).

### Osteohistology

**Figure 27:**
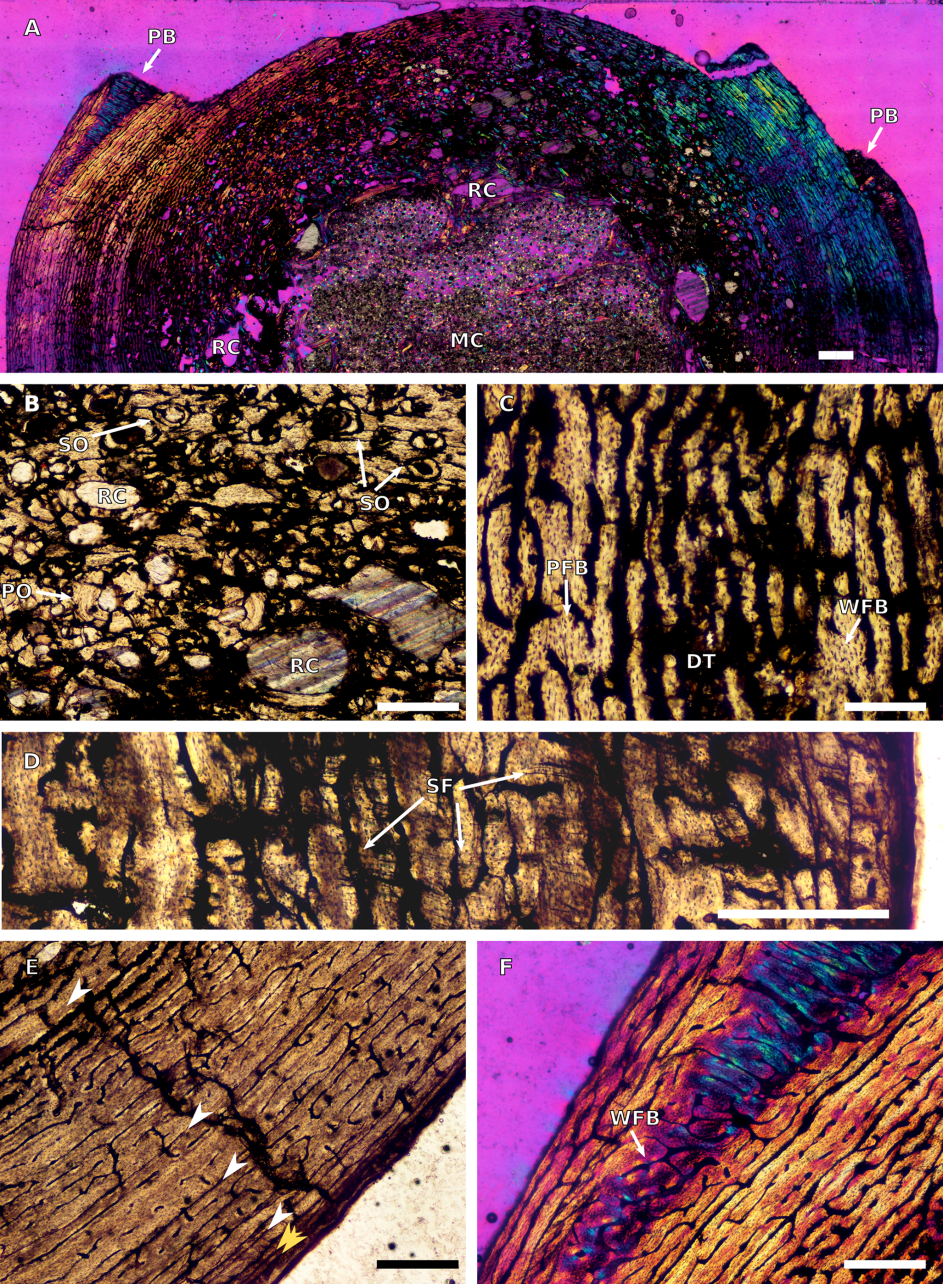
Humeral osteohistology of BP/1/4779. (A) Overall view in cross-polarized light of half the cross section, showing an open medullary cavity and large peri-medullary resorption cavities. PB indicates the location and extensity of pathological bone. (B) Close-up in normal light of the inner cortex, showing numerous resorption cavities and secondary osteons. (C) Close-up in normal light of the mid-cortex, showing a mixture of woven and parallel-fibred bone. (D) Close-up in normal light of the outer cortex, showing numerous Sharpey’s fibres. (E) Close-up in normal light of the outer cortex, showing decreased spacing between growth marks towards the sub-periosteal surface, but no EFS. (F) Close-up in cross-polarized light of the pathological bone. Scale bars represent 1,000 μm in (A) and 500 μm in (B–F). Yellow arrowheads indicate double LAGs; white arrowheads indicate single LAGs. Abbreviations: DT, diagenetic bone tissue; MC, medullary cavity; PB, pathological bone; PFB, parallel-fibred bone; PO, primary osteon; RC, resorption cavity; SF, Sharpey’s fibres; SO, secondary osteon; WFB, woven-fibred bone.

A section was taken from the middle of the right humeral midshaft. A relatively thin cortex surrounds an open medullary cavity (Fig. 27A). At its thickest, the cortex including spongy bone represents 60% of the thickness of the section (from the centre of the section to the sub-periosteal surface). Several large resorption cavities line the medullary cavity (Fig. 27B), with numerous smaller cavities extending into the mid-cortex. Scattered secondary osteons are present in the inner cortex, but do not form dense Haversian bone. Regions of the inner and mid-cortex are diagenetically altered and numerous cracks give the inner cortex a fragmentary appearance. However, small patches of interstitial matrix can still be seen and comprise parallel-fibred bone with evenly distributed globular and flattened osteocyte lacunae.

The preserved vascular canals in the mid-cortex are elongate and circumferentially oriented although this is difficult to confirm due to diagenesis and numerous cracks. The interstitial matrix of the mid-cortex is mainly parallel-fibred bone with some scattered patches of woven-fibred bone (identified by the presence of plentiful, disorganized and globular osteocyte lacunae) (Fig. 27C). There are no secondary osteons present in the mid-cortex.

The outer cortex is missing its anterior portion. The osteocyte lacunae become progressively more flattened and organized towards the sub-periosteal surface, although patches of woven bone matrix can still be seen. However, the bone tissue is predominantly a mixture of lamellar and parallel-fibred bone tissues. There is a decrease in vascularization from the mid-cortex to the outer cortex. The vascular arrangement varies between circumferentially-oriented and longitudinally-oriented primary osteons with short anastomoses. Sharpey’s fibres are visible in the outer cortex, but only in a small area on the posteromedial side (Fig. 27D).

The cracks make it difficult to determine an accurate number of lines of arrested growth (LAGs), but six LAGs could be confidently identified in the humerus. These correspond to periodic, but temporary cessations in growth. An external fundamental system (EFS), consisting of avascular lamellar bone with multiple, closely-spaced LAGs at the sub-periosteal surface and that would indicate the attainment of maximum size, was not observed (Fig. 27E).

There is a section of pathological bone tissue in the outer cortex, on either side of the missing anterior cortical surface. This tissue comprises very densely packed, disorganized and globular osteocyte lacunae with primary osteons arranged either radially or in a reticular network (Fig. 27F). The tissue tapers out at approximately 2,300 μm beneath the missing cortical surface. It appears to be localized and could not be located in the left humerus.

**Figure 28:**
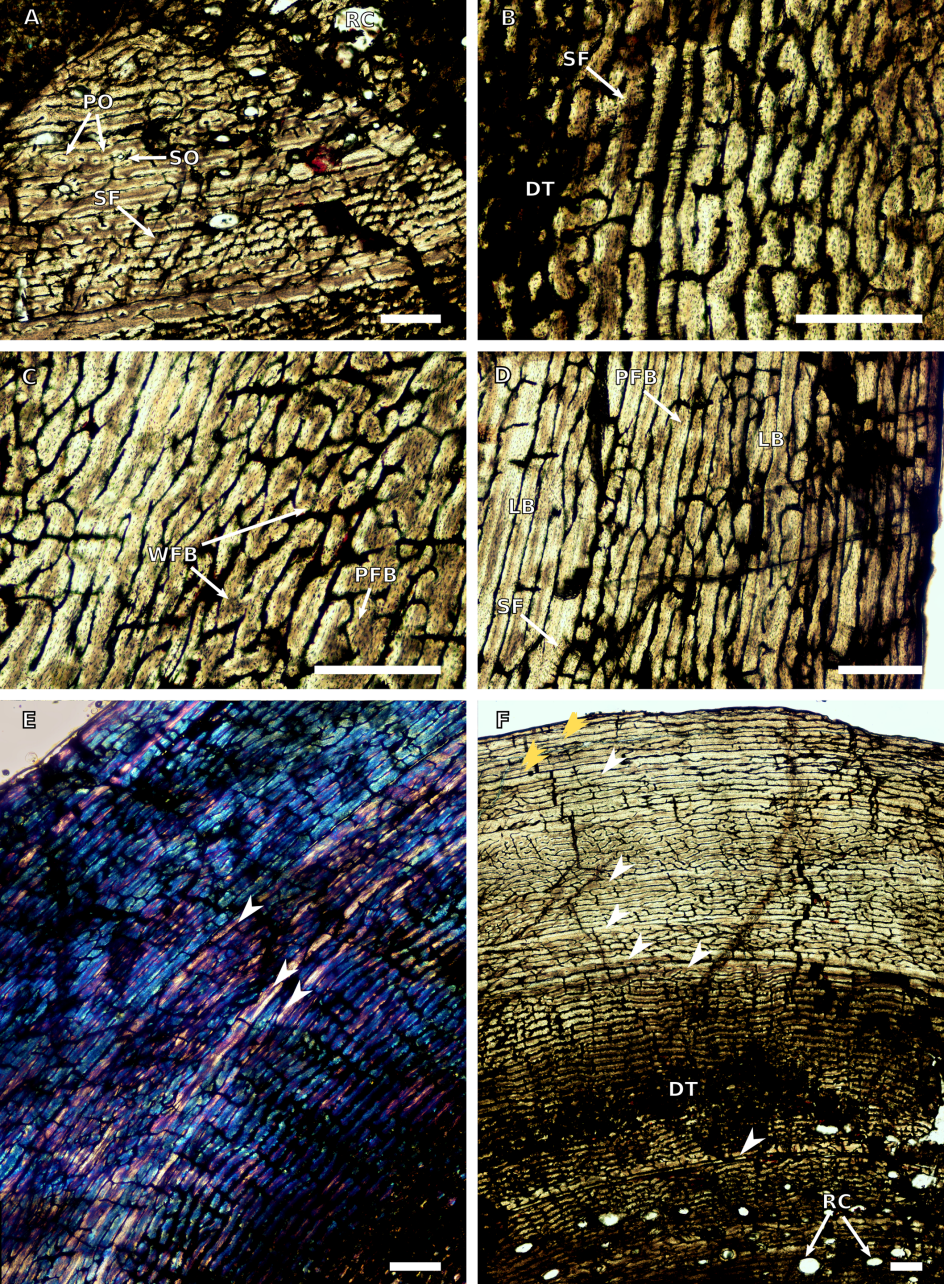
Femoral osteohistology of BP/1/4779. (A) Close-up in normal light of the inner cortex, showing several resorption cavities. (B) Close-up in normal light of the inner cortex, showing the diagenetic bone tissue. (C) Close-up in normal light of the mid-cortex, showing a mixture of woven and parallel-fibred bone tissue. (D) Close-up in normal light of the outer cortex, showing slowly forming lamellar bone. (E) Inner to outer cortex in cross polarised light, showing several growth marks. (F) Inner to outer cortex in normal light, showing double LAGs at the sub-periosteal surface. Scale bars represent 100 μm in (A) and 500 μm in (B–F). Yellow arrowheads indicate double LAGs; white arrowheads indicate single LAGs. Abbreviations: DT, diagenetic bone tissue; LB, lamellar bone; PFB, parallel-fibred bone; PO, primary osteon; RC, resorption cavity; SF, Sharpey’s fibres; SO, secondary osteon; WFB, woven-fibred bone.

Another section was taken from the middle of the left femoral midshaft. At its thickest, the cortex represents 61% of the section thickness (from the centre of the section to the sub-periosteal surface). The medullary cavity is open and lined with resorption cavities along its anterior and posterolateral margins (Fig. 28A). These resorption cavities decrease in size and become scattered towards the mid-cortex. A thick band between the inner and mid-cortex is diagenetically altered (Fig. 28B), obstructing our view of most of the interstitial tissue.

Some scattered primary osteons can be seen in the inner cortex, with a higher number in the posterolateral corner (Fig. 28C). Very few secondary osteons can be seen in the inner cortex, apart from a few isolated ones in the anterior and lateral portions of the section. The interstitial matrix of the inner cortex is mainly parallel-fibred with some patches of woven bone. It is therefore a mix of parallel fibred and fibrolamellar bone. The vascular canals in the inner cortex form a plexiform arrangement. Sharpey’s fibres extend to the inner cortex along the posterior side of the section.

The mid-cortex is also a mix of parallel-fibred and woven bone, although there appears to be an increase in woven bone patches compared to the inner cortex. Annuli, which indicate a temporary decrease in growth rate, consist of lamellar bone and interrupt the faster growing bone tissue. The vascular canals alternate between plexiform and laminar arrangements.

The outer cortex comprises parallel-fibred and lamellar bone (Fig. 28D). The vascular arrangement is mostly laminar although some areas form a plexiform network as well. There is also a decrease in vascularisation towards the sub-periosteal surface. Sharpey’s fibres can be seen in the outer cortex in areas around the section. Several annuli comprising slowly forming lamellar bone are observed throughout the cortex. They are more visible in CPL light in the mid and outer cortex (Fig. 28E).

Lines of arrested growth are present in the outer cortex and are more easily identified in CPL light. They are associated with annuli of lamellar bone. They become more closely spaced towards the sub-periosteal surface forming double LAGs in places, indicating a decrease in growth rate (Fig. 28F). In total 10 growth marks were counted throughout the cortex. No EFS was observed in the femur.

### Phylogenetic analysis

When compared to adult *M. carinatus* specimens BP/1/5241 and BP/1/4934, *N. intloko* differs in 25 character scores (Table 2). Due to slight ontogenetic variation, only the character scores common to the two *M. carinatus* specimens were compared. Characters that could only be scored in one *M. carinatus* specimen were also compared.

**Table 2:** Differing character scores between *M. carinatus* and *Ngwevu intloko*.

Character number	Character description	*Ngwevu intloko*	*Massospondylus carinatus*
2	Premaxilla: ventrolateral margin of alveolar region extends further ventrally than ventromedial margin in anterior/posterior view	Present	Absent
5	Premaxilla: morphology of the distal end of the nasal ramus (dorsal ramus) of the premaxilla	Tapered	Mediolaterally expanded
6	Premaxilla: lateral surface of the premaxilla	With an inflection at the base of the nasal ramus (dorsal process)	Convex
35	Antorbital fossa: shape of the antorbital fossa	Crescentic with a strongly concave posterior margin that is roughly parallel to the rostral margin of the antorbital fossa	Subtriangular with a straight to gently concave posterior margin
49	Prefrontal: maximum transverse width of the prefrontal	Less than 0.25 of the skull width at that level	More than 0.25 of the skull width at that level (BP/1/5241)
56	Postorbital: mediolateral width of the jugal ramus (ventral ramus) of the postorbital	Less than its anteroposterior width at midshaft	Greater than its anteroposterior width at midshaft
58	Postorbital: distal end of frontal process, distinct concave notch between parietal and frontal facets	Present	Absent
63	Frontal: presence of anterior portion of supratemporal fossa on posterior end of dorsal surface of frontal	Deeply excavated, forming a scarp- like margin	Weak
64	Supratemporal fenestra: orientation of the long axis	Transverse	Longitudinal
69	Quadratojugal: angle of divergence between jugal and squamosal rami of quadratojugal in lateral view	Close to parallel	Close to 90°
73	Quadrate foramen: position of the quadrate foramen	Deeply incised into, and partly encircled by, the quadrate	On the quadrate-quadratojugal suture
82	Supraoccipital: shape of the supraoccipital in posterior view	Semilunate and wider than high	Diamond-shaped, at least as high as wide
90	Basisphenoid: angle separating the long axes of the basiperygoid processes in anterior view	More than 60°	60° or less
96	Basisphenoid: orientation of basipterygoid processes long axes in lateral view	Extend posteroventrally	Extend ventrally or near ventrally
100	Basisphenoid: orientation of main body (axis passing through middle of posterior margin of basal tubera and junction between base of basipterygoid process and cultriform process) in lateral view	Slopes anteroventrally	Anteroposteriorly oriented, horizontal
102	Laterosphenoid: orientation of postorbital ramus	Extends anterodorsally	Extends laterally
103	Laterosphenoid: orientation of frontal ramus	Extends medially	Extends anteromedially
104	Basioccipital: ventral margin of basioccipital condyle	Dorsal to proximal base of basipterygoid processes	Aligned with or ventral to proximal base of basipterygoid processes
109	Palatine: position of the maxillary articular surface of the palatine	Along the lateral margin of the bone	At the end of a narrow anterolateral process due to the absence of the posterolateral process (BP/1/5241)
113	Vomer: length	Less than 0.25 of the total skull length	More than 0.25 of the total skull length (BP/1/5241)
115	Jaw: shape of articulated premaxillae and maxillae in ventral view	Broad and ‘U’-shaped	Narrow with an acute rostral apex
132	Teeth: orientation of the dentary tooth crowns	Procumbent	Erect (BP/1/4934)
168	Number of vertebrae between cervicodorsal transition and primordial sacral vertebrae	No more than 14	15–16 (BP/1/4934)
232	Well-defined fossa on the distal flexor surface of the humerus	Absent	Present
318	Position of fourth trochanter along the mediolateral axis of the femur	On the medial margin	Centrally located (BP/1/4934)

**Note:**

Character scores for *M. carinatus* are those common to BP/1/5241 and BP/1/4934 except where specimen number specified, indicating that the character could not be scored in one of the specimens.

**Figure 29:**
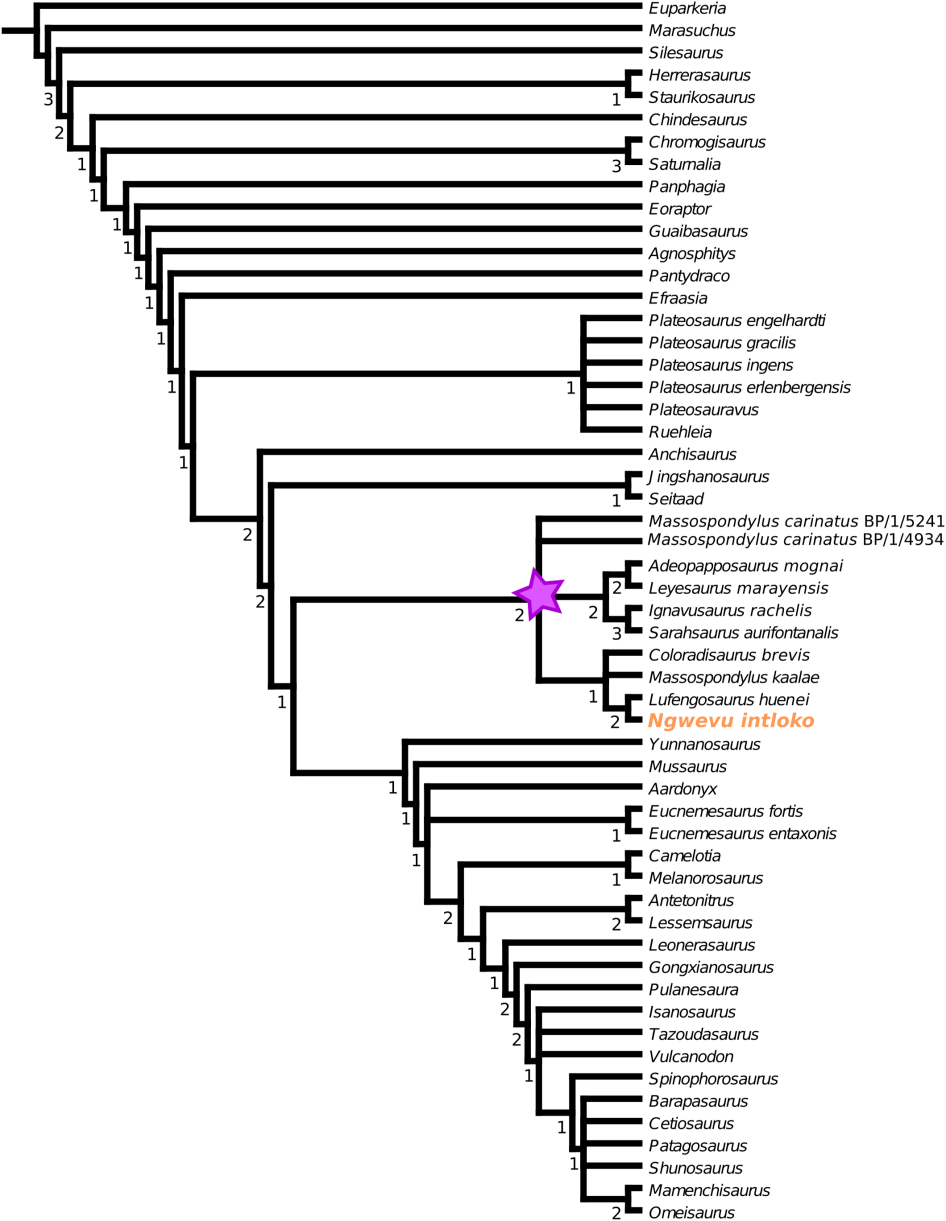
Strict consensus tree of 120 most parsimonious trees of 1,269 steps, a consistency index of 0.340 and a retention index of 0.630. The purple star indicates Massospondylidae clade. Numbers indicate Bremer support for each node.

The New Technology Search yielded 60 most parsimonious trees (MPTs) with lengths of 1,269 steps. Additional TBR swapping on the MPTs from the new technology search yielded a total of 120 MPTs with tree lengths of 1,269 steps, a Consistency Index of 0.340 and a Retention Index of 0.630 (see File S2 for matrix). The strict consensus tree of these MPTs is presented in Fig. 29.

All of the trees place *Ngwevu* within a monophyletic Massospondylidae that also includes *Sarahsaurus aurifontanalis*, *Ignavusaurus rachelis*, *Leyesaurus marayensis*, *Adeopapposaurus mognai, M. carinatus* (BP/1/5241 and BP/1/4934), *Coloradisaurus brevis*, *M. kaalae* and *Lufengosaurus huenei*. This clade is supported by one postcranial unambiguous synapomorphy: lateral margins of the pubic apron concave in lateral view (character 286, state 1). It is also supported by 11 ambiguous cranial synapomorphies and one ambiguous postcranial synapomorphy. The cranial synapomorphies consist of: depression behind the naris (character 22, state 1); antorbital fossa that reaches the anterior tip of the jugal (character 31, state 0); mediolateral width of the jugal ramus of the postorbital is greater than its anteroposterior length at midshaft (character 56, state 1); jugal contacts the lateral or dorsal surface of the quadratojugal (character 72, states 0 and 2); pterygoid ramus of the quadrate occupies <0.7 the total height of the quadrate (character 76, state 0); supraoccipital dorsal margin strongly sloping forward so that the dorsal tip lies level with the basipterygoid processes (character 83, state 1); angle separating the long axes of the basiperygoid processes is more than 60° (character 90, state 0); ventral margin of the basal tubera lie ventral to the proximal base of the basipterygoid processes (character 98, state 1); basisphenoid main body is anteroposteriorly oriented (character 100, state 0); absence of a pneumatic fossa on the ventral surface of the ectopterygoid (character 107, state 1); and dentary curves ventrally (character 119, state 1). The postcranial synapomorphy is the relative elongation of the cervical centra with the length of at least cervical 4 or 5 exceeding four times the anterior centrum height (character 153, state 2).

In all of the MPTs and in the strict consensus tree, *Ngwevu* and *Lufengosaurus huenei* are sister taxa. This relationship is supported by seven ambiguous cranial synapomorphies and one ambiguous postcranial synapomorphy. These synapomorphies are: ventrolateral margin of the premaxilla extends further than the ventromedial margin (character 2, state 1); the development of the antorbital fossa on the lacrimal ramus of the maxilla is weakly impressed and delimited by a rounded rim (character 33, state 1); crescentic antorbital fossa (character 35, state 0); deeply incised supratemporal fossa on the frontal (character 63, state 1); supratemporal fenestra that is mediolaterally wider than it is anteroposteriorly long (character 64, state 1); quadratojugal ramus of the squamosal is not four times as long as it is wide at the base (character 65, state 0); the angle separating the basipterygoid processes is more than 60° (character 90, state 1) and angle between the long axis of the femoral head and the distal femur is close to 0° (character 304, state 1).

A more inclusive monophyletic group including the latter clade, *Coloradisaurus* and *M. kaalae* is also recovered, but its interrelationships are not resolved. This clade is supported by 14 ambiguous synapomorphies: the mediolateral width of the jugal ramus (ventral ramus) of the postorbital is less than its anteroposterior length at midshaft (character 56, state 0); the angle of divergence between jugal and squamosal rami of quadratojugal in lateral view is close to parallel (character 69, state 1); the quadrate foramen is deeply incised into, and partly encircled by the quadrate (character 73, state 1); the long axis of the basipterygoid processes extends posteroventrally in lateral view (character 96, state 2); the ventral margins of the basal tubera are level or dorsal to the proximal base of the basipterygoid processes in lateral view (character 98, state 0); the main body of the basisphenoid (axis passing through middle of the posterior margin of basal tubera and junction between base of basipterygoid process and cultriform process) slopes anteroventrally in lateral view (character 100, state 1); the ventral margin of the basioccipital condyle is dorsal to the proximal base of the basipterygoid processes in lateral view (character 104, state 1); the anteroposterior length of the retroarticular process is greater than the depth of the mandible below the glenoid (character 126, state 1); the texture of the tooth enamel surface is entirely smooth (character 139, state 0); the anteroposterior expansion of the distal pubis is greater than 0.15 of the length of the pubis (character 289, state 2); the profile of the fourth trochanter of the femur is symmetrical, almost rectangular in lateral view with the proximal and distal corners approaching an angle of almost 0 (character 316, state 2); the distal surface of the tibiofibular crest is mediolaterally wider than anteroposteriorly long (character 322, state 1); the presence of a well-developed facet on the proximolateral corner of the plantar ventrolateral flange of MTII for articulation with the medial distal tarsal (character 363, state 1); and the proximal outline of MTIII is subtrapezoidal with the posterior margin broadly exposed in plantar view (character 365, state 1).

In the strict consensus tree, the two adult *M. carinatus* specimens form a polytomy outside of the clade containing *Ngwevu*, *Lufengosaurus huenei*, *Coloradisaurus brevis* and *M. kaalae* and that contains *Sarahsaurus*, *Ignavusaurus*, *Leyesaurus* and *Adeopapposaurus*. These two *M. carinatus* specimens have inconsistent positions within the MPTs and shift between the two clades. In some topologies, BP/1/5241 is sister taxon to the more derived monophyletic clade containing BP/1/4934, *Coloradisaurus*, *M. kaalae*, *Ngwevu* and *Lufengosaurus huenei*. In other topologies, BP/1/4934 and BP/1/5241 form a monophyletic clade that is the sister taxon of a larger clade comprising *Sarahsaurus*, *Ignavusaurus*, *Leyesaurus* and *Adeopapposaurus*.

## Discussion

In the first half of the 20th century, the genus *Massospondylus* was subjected to intensive splitting (Seeley, 1895; Broom, 1911; Haughton, 1924) but the focused revision of Cooper (1981) synonymized many of these species with *M. carinatus*. It is therefore imperative that great caution be taken when establishing any new massospondylid taxon. Here, we examine other possible explanations for the morphological differences documented above, including ontogeny, sexual dimorphism and distortion.

Gow, Kitching & Raath, (1990) and Sues et al. (2004) referred BP/1/4779 to *M. carinatus*, regarding the differences in skull proportions between it and other referred specimens as the result of taphonomic deformation (oblique dorsoventral crushing and anteroposterior compression). In general, two types of taphonomic deformation are prevalent in the fossil record, brittle and plastic deformation, and definitive criteria have been laid out for each (Lautenschlager, 2017). Using these criteria, and our morphological observations, we reject the suggestion of Gow, Kitching & Raath, (1990) and Sues et al. (2004) based on three factors: (1) lack of evidence for extensive brittle deformation; (2) lack of evidence for extensive plastic deformation; and (3) morphological differences between BP/1/4779 and individuals referred to *M. carinatus*.

When brittle deformation occurs, the specimen will display cracks, breaks and fragmentation (Lautenschlager, 2017) and some degree of disarticulation could also be expected. Although some portions of the bones are missing in BP/1/4779, these appear to have been eroded (such as the thin nasal and the lateral surface of the alveolar region of the premaxilla) and the number of large, external cracks is minimal. The specimen is also well articulated with minimal disruption to the skeleton.

Plastic deformation can be identified by the loss of bilateral symmetry without disarticulation, for example the deformation of the orbit into an oval rather than circular shape that maintains bone-on-bone contacts around its periphery. BP/1/4779 has a noticeably symmetrical skull, with only a slightly sub-circular orbit. CT scans reveal that the orbital sub-circularity is due to the ventral displacement of the frontals, which gives the orbit a slightly dorsoventrally compressed appearance. There is no other evidence of such distortion in any other part of the skeleton and it seems unlikely that the skull could suffer plastic deformation while other nearby elements, such as the Cv, were unaffected.

Finally, BP/1/4779 has proportionally robust cranial bones and discrete morphological differences when compared to *M. carinatus* that cannot be explained by taphonomic distortion. These include: the proportions of the supratemporal fenestrae (being mediolaterally wider than they are anteroposteriorly long); the anteromedial process of the maxilla extending anteromedially and the overall concave medial and convex lateral margins of the maxilla; the proportionally dorsoventrally high frontal ramus of the prefrontal; the rounded lacrimal angle; the anterior flaring of the lacrimal shaft; the crescentic antorbital fenestra; the pronouncedly robust postorbital; the ridge on the lateral surface of the jugal; the semicircular pterygoid and quadratojugal rami of the quadrate; the robust ventral portion of the quadrate with a convex anterior margin; the morphology of the vomers; the dorsolateral orientation of the postorbital ramus of the laterosphenoid; the elongation and angle of the posterodorsal portion of the prootic; the robustness of the basisphenoid; the dorsoventrally compressed basisphenoid body but dorsoventrally high cultriform process; the wide angle separating the basal tuberae; the wider angles separating the paroccipital processes of the exoccipitals as well as the squamosal rami of the parietals; the dorsoventrally high coronoid region of the mandible; the proportionally gracile thumb; and the lack of a dorsosacral vertebra.

Although taphonomic deformation could be responsible for some of the overall orientation differences observed in the skull (notably the posteroventral sloping of the posterior portion of the skull), the shape variation of individual bones, the skull proportions, and the robustness of the bones are not explained by taphonomic deformation. In order for taphonomy to be wholly responsible for the differences observed, deformation would have to be in mutually exclusive directions, which is unlikely.

A well-documented ontogenetic series is known for *M. carinatus*, including three well-preserved skulls that together bracket the size of BP/1/4779. These specimens, BP/1/4376, BP/1/5241 and BP/1/4934, show high levels of morphological similarity (Gow, 1990; Gow, Kitching & Raath, 1990; Sues et al., 2004; Chapelle & Choiniere, 2018). BP/1/4779 possesses features not seen in any of these specimens (see above), and many of these do not appear to be under ontogenetic control as they are not seen to change within the *M. carinatus* ontogenetic series. For example, the majority of skull proportions remain consistent in BP/1/4376, BP/1/5241 and BP/1/4934 and the extreme widening and foreshortening of the skull seen in BP/1/4779 is not hinted at in any of these other individuals. To invoke ontogeny as the factor leading to these differences a scenario would have to be envisaged in which the skull began with the elongate, narrow form seen in BP/1/4376, which would then have to expand markedly laterally in BP/1/4779, before narrowing once more to the condition in BP/1/4934 and BP/1/5421. Such major, fluctuating changes in cranial proportions through ontogeny seem highly unlikely.

Based on histological analysis, BP/1/4779 reached a minimum age of 10 years and was nearly fully grown at the time of death, based on the number of LAGs and the decreasing space between them. Although it has been hypothesized that some basal sauropodomorph dinosaurs, like *Plateosaurus*, displayed developmental plasticity and variable life histories, potentially in response to seasonal environmental changes (Sander & Klein, 2005), no evidence of this plasticity has been recorded in *Massospondylus* thus far (Chinsamy, 1993).

Finally, an independent line of supporting evidence comes from the inner ear morphology of BP/1/4779 (Neenan et al., 2018).This study investigated the ontogeny of vestibular canal shape and size in *M. carinatus* and found that BP/1/4779 did not follow the shape trajectory seen in the other specimens, but was an outlier whose vestibular canal shape could not be explained by ontogenetic trajectory alone. All of these lines of evidence lead us to reject the hypothesis that BP/1/4779 is a juvenile *M. carinatus* (*contra* Gow, Kitching & Raath, 1990; Sues et al., 2004).

An alternative hypothesis is that sexual dimorphism might account for the differences between BP/1/4779 and other skulls referred to *M. carinatus*. Among living animals, many sexually dimorphic characters are restricted to soft tissue anatomy, behaviour or differences in body size and colouration, but there are examples of skeletal differences also, such as the presence/absence or size and shape of horns, crests or frills (Chapman, 1997; Barden & Maidment, 2011). In extinct taxa, sexual dimorphism is often difficult to confirm due to the lack of preservation of these elements, ambiguities in sexing individual skeletons and inadequate sample sizes. In fact, recent studies using statistical methods, such as unimodality and normality tests, on morphological data found no evidence for sexual dimorphism in any of the nine dinosaur species examined. These species included theropods (*Coelophysis bauri*, *Coelophysis rhodesiensis*, *Allosaurus fragilis*, *Tyrannosaurus rex*), sauropodomorphs (*Plateosaurus* spp.), and ornithischians (*Stegosaurus mjosi*, *Kentrosaurus aethiopicus*, *Stegoceras validum*, *Protoceratops andrewsi*), all of which have been hypothesized to display sexual dimorphism in previous research (Mallon, 2017). In order to distinguish between sexes morphologically, they have to be identified beforehand through other means, such as the presence of eggs, embryos, biomechanical structures and differences in histological data (Mallon, 2017). Even in cases where an apparently clear sexual difference has been identified the reliability of these features have been questioned. For example, a recent study found that the presence of medullary-like bone in non-avian archosauromorphs that laid soft-shelled eggs or were not yet reproductively mature, challenged the long standing thought that these medullary like tissues are strictly associated with the production of eggshell (Prondvai, 2017). Ascertaining the sex of a fossil individual is therefore tenuous and ideally requires large sample sizes as well as unambiguous evidence.

BP/1/4779 is a smaller individual than adult *M.* carinatus specimens (BP/1/4934, BP/1/5421) and possesses relatively robust cranial bones. Although these differences in size and robusticity could be due to sexual dimorphism, the other morphological differences that are present are unlikely to be the product of sexual dimorphism (including shape differences between bones that do not have clear display or mating structures, such as the structure of the braincase and palate).

Many of the morphologies that distinguish *Ngwevu* from *M. carinatus* are related to feeding or jaw muscle attachment. For example, the well-developed coronoid eminence, mediolaterally wide palate, short snout and fused skull roof bones may have allowed for larger muscle mass and stronger bite force. Among sauropodomorphs, it has been hypothesized that mediolaterally narrow snouts are more efficient for selective browsing as opposed to mediolaterally wider snouts that are suited for generalist bulk browsing (Upchurch, Barrett & Galton, 2007). In comparison to *M. carinatus*, BP/1/4779 has a proportionally wider snout that might indicate an ecological difference between these sympatric taxa, which could have been important in allowing niche partitioning between animals that were similar in overall form and body size.

*Ngwevu* is from the upper Elliot Formation, which preserves numerous contemporaneous basal sauropodomorphs and represents deposition over a period of approximately five million years. Consequently, another hypothesis that should be considered to account for the differences between it and other specimens is anagenetic change, as species do not generally persist for such long intervals (Scannella et al., 2014). However, in order to confirm anagenesis, it is necessary to have high-resolution stratigraphy in order to identify evolutionary trends (Scannella et al., 2014). Based on our phylogenetic hypothesis, which does not recover *Ngwevu* as a close relative of other upper Elliot sauropodomorphs, anagenesis seems unlikely. Modern generalist herbivore communities are often relatively diverse in the low-to-medium body size category (Du Toit & Cumming, 1999) and this could also be true of Mesozoic herbivore communities.

Although no other specimens can be referred to *Ngwevu* at present, SAM-PK-K1314 (referred to *M. carinatus*) shares similar skull proportions and some similar cranial morphologies (Barrett & Yates, 2005). However, due to the clear dorsoventral crushing of this skull, further investigation is necessary.

Massospondylidae was a biogeographically widespread clade, with members on three continents at times ranging from the Late Triassic to the Early Jurassic. Throughout massospondylid evolutionary history Pangaea remained intact, so perhaps unsurprisingly our phylogenetic analysis does not identify any endemic, geographically restricted subclades. Our strict consensus tree recovers a clade comprising *Coloradisaurus* from South America, *M. kaalae* and *Ngwevu* from southern Africa and *Lufengosaurus huenei* from China. This larger clade is, in turn, the sister group of a clade comprising *Ignavusaurus* from southern Africa and *Sarahsaurus* from North America. One minor exception to this is the sister-taxon relationship found between *Leyesaurus* and *Adeopapposaurus*, which are both South American.

Understanding palaeobiogeographical and evolutionary patterns requires a robust phylogenetic hypothesis. In basal sauropodomorphs, this is hindered by the incompleteness of the fossil record, the available sample sizes for many taxa as well as the completeness of character matrices used in analyses. The latter is especially true for Massospondylidae, which is a poorly resolved clade that has been resolved into many different topologies depending on the characters and taxa included in respective analyses. Although massospondylid remains are fairly numerous, the character matrices used to resolve early branching sauropodomorphs lack sufficient homology hypotheses for robustly resolving higher level relationships. For example, a combination of 22 characters distinguishes BP/1/4779 from other massospondylid taxa, but many of these are not included in existing character matrices. Some of these characters cannot be scored in some taxa due to specimen incompleteness or lack of preparation. This work illustrates the importance of CT scanning in expanding anatomical datasets, and therefore the potential for identifying new, useful characters and character states for further detailed analyses and also for increasing the sample sizes of included specimens. It is likely that we will only obtain a better understanding of massospondylid ingroup relationships (and indeed relationships in some other areas of the sauropodomorph tree) if we are able to gather more information in this way from both new and historically collected specimens. Furthermore, in the case of massospondylids, identifications to species-level usually require the discovery of reasonably complete skeletons, as many of these taxa are diagnosed by suites of characters that are distributed throughout the skeleton (and that exhibit at least some homoplasy) rather than by unambiguous autapomorphies. It will be necessary, therefore, to re-assess specimens that have already been accessioned in order to identify them more robustly. The historical practice of referring incomplete specimens to specific massospondylid taxa should be abandoned unless there is a firm basis for such referrals (see also Barrett et al., 2019). Using this more rigorous approach to identification will facilitate better understanding of the diversity and biostratigraphy of the Late Triassic–Early Jurassic.

## Conclusion

Although previously referred to *Massospondylus carinatus*, the holotype specimen of *Ngwevu intloko* can be differentiated from other basal sauropodomorphs by a combination of 22 characters. These characters are not the result of ontogeny, sexual dimorphism or distortion. Phylogenetic analysis reveals that Massospondylidae was a diverse, successful clade with members present on three major continents between the Late Triassic and Early Jurassic. The description of *Ngwevu* shows the utility of better understanding ontogenetic variation in densely sampled dinosaur taxa and the benefits of critically re-assessing previously collected material.

## Supplemental Information

10.7717/peerj.7240/supp-1 Supplemental Information 1 Table listing specimen numbers of specimens used for comparative purposes as well as references and collections where housed.

10.7717/peerj.7240/supp-2 Supplemental Information 2 Character matrix used and trees generated by phylogenetic analysis.

## Institutional Abbreviations

BP: Evolutionary Studies Institute, University of the Witwatersrand, Johannesburg, South Africa
SAM: Iziko South African Museum, Cape Town, South Africa.

## Additional Information and Declarations

### Data Availability

The following information was supplied regarding data availability: Raw data are available at Morphosource: https://www.morphosource.org/Detail/ProjectDetail/Show/project_id/735 and http://www.morphosource.org/Detail/ProjectDetail/Show/project_id/426. The raw data consist of .stl format files of BP/1/4779 and BP/1/4376 (the skulls and individual bones presented in this article) and BP/1/5241 scans (DICOM stack). A request to access the data will be sent to the Evolutionary Studies Institute Curator who will approve all bona fide requests.

### New Species Registration

The following information was supplied regarding the registration of a newly described species: Publication LSID: urn:lsid:zoobank.org:pub:839E7CB8-82D6-47FF-B348-43ED9106D995. *Ngwevu intloko* gen. & sp. nov. LSID: urn:lsid:zoobank.org:act:4798B77D-BD14-4AA8-86E5-FE45476874E5.
