# Supplementary material for: Ngwevu intloko: a new early sauropodomorph dinosaur from the Lower Jurassic Elliot Formation of South Africa and comments on cranial ontogeny in Massospondylus carinatus
Source: PeerJ. 2019 Aug 5;7:e7240. doi: 10.7717/peerj.7240 (PMC6687053; doi:10.7717/peerj.7240)
Supplement: Supplemental Information 1 [file peerj-07-7240-s001.docx]

| Taxon | Observations and scores based on | Specimen number | Location of specimen |
| --- | --- | --- | --- |
| *Adeopapposaurus mognai* | Martínez 2009 | PVSJ610 and PVSJ568 | Instituto y Museo de Ciencias Naturales, Universidad  Nacional de San Juan, San Juan, Argentina |
| *Arcusaurus pereirabdalorum* | Yates et al. 2011  Personal specimen examination | BP/1/6235 | Evolutionary Studies Institute, University of the Witwatersrand, Johannesburg, South Africa |
| *Coloradisaurus brevis* | Apaldetti et al. 2014 | PVL 3967, holotype | Instituto Miguel Lillo, Universidad de Tucuman, Tucuman, Argentina |
| *Ignavusaurus rachelis* | Knoll 2010 | BM HR 20 | Lesotho National Museum, Maseru, Lesotho |
| *Leyesaurus marayensis* | Apaldetti et al. 2011 | PVSJ 706, holotype | Instituto y Museo de Ciencias Naturales, Universidad  Nacional de San Juan, San Juan, Argentina |
| *Lufengosaurus huenei* | Barrett et al. 2005  Personal specimen examination | IVPP V15 | Institute of Vertebrate Palaeontology, Beijing, China |
| *Massospondylus kaalae* | Barrett 2009  Personal specimen examination | SAMPK-K1325, holotype | Iziko South African Museum, Cape Town, South Africa |
| *Sarahsaurus aurifontanalis* | Marsh & Rowe 2018  Personal specimen examination | MCZ 8893 | Museum of Comparative Zoology, Harvard University, Boston, United States of America |
